# A decade of dinoflagellate genomics illuminating an enigmatic eukaryote cell

**DOI:** 10.1186/s12864-024-10847-5

**Published:** 2024-10-04

**Authors:** Senjie Lin

**Affiliations:** Department of Marine Sciences, University of Connecticut, Groton, CT 06340 USA

**Keywords:** Dinoflagellate, Genome expansion, Omics, Nuclear protein, Histone, Gene regulation, Retroposition, MicroRNA (miRNA), Parasitic, Symbiotic

## Abstract

Dinoflagellates are a remarkable group of protists, not only for their association with harmful algal blooms and coral reefs but also for their numerous characteristics deviating from the rules of eukaryotic biology. Genome research on dinoflagellates has lagged due to their immense genome sizes in most species (~ 1-250 Gbp). Nevertheless, the last decade marked a fruitful era of dinoflagellate genomics, with 27 genomes sequenced and many insights attained. This review aims to synthesize information from these genomes, along with other omic data, to reflect on where we are now in understanding dinoflagellates and where we are heading in the future. The most notable insights from the decade-long genomics work include: (1) dinoflagellate genomes have been expanded in multiple times independently, probably by a combination of rampant retroposition, accumulation of repetitive DNA, and genome duplication; (2) Symbiodiniacean genomes are highly divergent, but share about 3,445 core unigenes concentrated in 219 KEGG pathways; (3) Most dinoflagellate genes are encoded unidirectionally and are not intron-poor; (4) The dinoflagellate nucleus has undergone extreme evolutionary changes, including complete or nearly complete loss of nucleosome and histone H1, and acquisition of dinoflagellate viral nuclear protein (DVNP); (5) Major basic nuclear protein (MBNP), histone-like protein (HLP), and bacterial HU-like protein (HCc) belong to the same protein family, and MBNP can be the unifying name; (6) Dinoflagellate gene expression is regulated by poorly understood mechanisms, but microRNA and other epigenetic mechanisms are likely important; (7) Over 50% of dinoflagellate genes are “dark” and their functions remain to be deciphered using functional genetics; (8) Initial insights into the genomic basis of parasitism and mutualism have emerged. The review then highlights functionally unique and interesting genes. Future research needs to obtain a finished genome, tackle large genomes, characterize the unknown genes, and develop a quantitative molecular ecological model for addressing ecological questions.

## Background

**Fig. 1:**
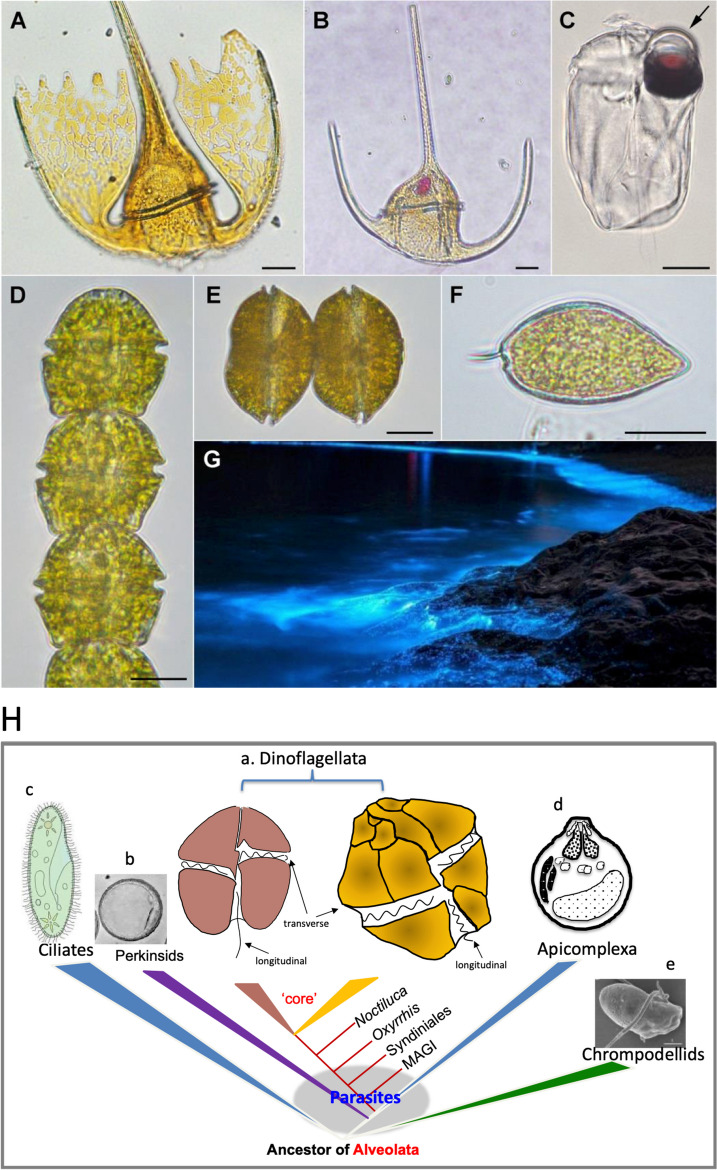
Light micrographs of dinoflagellates and a bioluminescence scene (A-G) and phylogenetic relationship of dinoflagellates, apicomplexa, and other alveolates (H). A. *Tripos platycorne*. B. *Tripos muelleri*. C. *Erythropsidinium agile*. The arrow points at the ocelloid. D. *Gymnodinium catenatum*. E. *Gessnerium* (=*Alexandrium*) *monilatum*. F. *Prorocentrum micans*. Scale bars = 20 µm. G. Bioluminescence by *Noctiluca scintillans* at Zhejiang, China. Images A-F courtesy of Dr. Fernando Gómez; all in apex facing up view except E and F being lateral view. Image G courtesy of Dr. Hao Luo. In H), core dinoflagellates (a) include naked dinoflagellate with two flagella (longitudinal and transverse) and thecate dinoflagellate with two flagella (longitudinal and transverse) and the conspicuous thecal plates. Non-core dinoflagellates include Marine Alveolate Group I (MAGI, euduboscquellids), Syndiniales (also known as MAGII), and the basal lineages *Oxyrrhis* and *Noctiluca*. Modified from Lin 2011 with up-to-date information

Dinoflagellates are one of the most important and unique groups of protists, notable for their ecological success and many peculiar characteristics that are a constant source of fascination [1, 2]. Generally believed to have emerged in the Silurian period (~ 400 MYA), the phylum of Dinoflagellata has over 2,300 extant species and roughly 2,000 fossil species [3, 4]. Morphologically, dinoflagellates are highly diverse (Fig. 1), but generally grouped into two major morphotypes: the thecate group in which cells contain cellulose-filled vesicles (amphiesma) underneath the cell membrane and the athecate (i.e. naked) group in which cells have weak or no discernible cellulose-filled amphiesma.

Ecologically, dinoflagellates are highly adaptable to vastly different habitats, from small ponds, rivers, lakes, and estuaries, to the ocean, and from the Antarctic to the Arctic, with lineages evolutionarily migrating between freshwater and marine environments [5, 6]. Many species in the Symbiodiniaceae are indispensable endosymbionts of reef-building corals. Many species from various lineages cause harmful algal blooms and produce toxins, devastating marine ecosystems and coastal economies as well as posing public health risks. Additionally, dinoflagellates live in diverse trophic modes (from autotrophic, heterotrophic, to mixotrophic) and lifestyles (from parasitic, symbiotic, to free-living), with photoautotrophic lineages (~ 50% of species) contributing substantially to photosynthetic carbon fixation and oxygen production and heterotrophic/mixotrophic species being important trophic links in the food chain. Dinoflagellates are the only group of protists that produce bioluminescence, which occurs in both photosynthetic (e.g. *Alexandrium* spp., *Lingulodinium polyedrum*) and heterotrophic or mixotrophic (e.g. red and green *Noctiluca scintillans*) species.

From an evolutionary perspective, dinoflagellates encompass parasitic (Syndiniales) lineages at the base followed by the typical dinoflagellate lineages composed of athecate and thecate lineages (Fig. 1), which have acquired various types of plastids originating from secondary, serial secondary, or tertiary endosymbiosis. At the cellular and genomic levels, dinoflagellates possess numerous peculiarities such as permanently (or nearly so) condensed chromosomes with cholesteric liquid crystalline state of DNA, lack of nucleosomes, closed mitosis, and extranuclear mitotic spindle [7]. Due to these characteristics, how dinoflagellates regulate DNA duplication in the cell division cycle and regulate gene expression in response to growth conditions remain to be demystified.

Due to the enormous genome sizes (up to 250 Gbp), genome sequencing for dinoflagellates has lagged behind many other algal phyla. However, thanks to the precipitous drop in sequencing costs and significant advances in bioinformatic methodology, dinoflagellate genome sequencing has finally taken off and reached a significant milestone over the past decade. Along with exponentially growing transcriptome and metatranscriptome data, the dinoflagellate -omic (Dinomic) data has grown to an appreciable database (Table 1). Although the database is strongly biased towards small dinoflagellate genomes, it contains rich information that illuminates various aspects of dinoflagellate biology. This wealth of data warrants a synthesis to bring the last reviews up to date [2, 8]. Some of the findings have challenged long-standing notions regarding dinoflagellate nuclear biology and molecular genetics, while others have revealed unsuspected physiologies. This review focuses on the most exciting discoveries from the last decade and anticipates what lies ahead for the next decade of Dinomic research.

**Table 1:** Published genomes of dinoflagellates

Species	Assembled genome size (Mbp)	Assembled rate (%)	Gene No.	Scaffolds N50 (kbp)	GC (%)	Repetitive DNA (%)	Reference
Amoebophrya sp. ex Karlodinium veneficum	~ 130	94.62	41,936	21	> 58		Bachvaroff 2019 [9]
Amoebophrya ceratii AT5.2	87.7	75.86	19,925	83.9	55.9	23.8	John et al. 2019 [10]
Amoebophrya ceratii A120	115.5	92.21	26,441	9,243	51.2	13.1	Farhat et al. 2021 [11]
Amoebophrya ceratii A25	116	97.8	28,091	1,082	47.8		Farhat et al. 2021 [11]
Amphidinium gibbosum	6,300	98.44	85,139	166.4	47.1	29.9	Beedessee et al. 2020 [12]
Breviolum minutum	616	41.07	41,925	126.2	43.6		Shoguchi et al. 2013 [13]
Cladocopium goreaui (original)	1,030	85.55	35,913	98	44.8		Liu et al. 2018 [14]
Cladocopium goreaui (improved)	1,200		45,322	354	44.4	36.5	Chen et al. 2022 [15]
Cladocopium spp.	704.8		65,832		43		Shoguchi et al. 2021 [16]
Durusdinium trenchii	670		30,054	97.5	47.4		Shoguchi et al. 2021 [16]
Effrenium voratum RCC1521	1,200	85.71	32,108	720	50.8		Shah et al. 2024 [17]
Effrenium voratum CCMP3420	1,300		39,878	252	50.6		Shah et al. 2024 [17]
Effrenium voratum CCMP421	1,100	57.89	32,615	304	50.9		Shah et al. 2024 [17]
Fugacium kawagutii CCMP2468	935	79.24	36,850	381			Lin et al. 2015 [18]
Fugacium kawagutii CCMP2468	937	79.41	45,192	13,533.5			Li et al. 2020 [19]
Fugacium kawagutii CCMP2468	1,050	88.98	26,609	268.8		16	Liu et al. 2018 [14]
Hematodinium sp.^a^	4,769		29,510	17,235			Gornik et al. 2015 [20]
Polarella glacialis CCMP1383	2,980		58,232	170.3	45.91	68	Stephens et al. 2020 [21]
Polarella glacialis CCMP2088	2,760		51,713	129.2	46.15	68	Stephens et al. 2020 [21]
Prorocentrum cordatum	4,750	87.37	85,849	349.2	59.7		Dougan et al. 2023 [22]
Symbiodinium linucheae CCMP2456	695	75.96		58.1	50.36		González-Pech et al. 2021 [23]
Symbiodinium microadriacticum CCMP2467	808	73.45	49,109	573.5	50.5	34.8	Aranda et al. 2016 [24]
Symbiodinium microadriacticum CassKB8	813	72.65		43	51.91		González-Pech et al. 2021 [23]
Symbiodinium microadriaticum 04-503SCI.03	775	73.62		50	50.46	27.9	González-Pech et al. 2021 [23]
Symbiodinium natans CCMP2548	762	100		610.5	51.79		González-Pech et al. 2021 [23]
Symbiodinium necroappetens CCMP2469	768	76.26		11.4	50.85		González-Pech et al. 2021 [23]
Symbiodinium pilosum CCMP2461	1,089	54.64		62.4	48.21		González-Pech et al. 2021 [23]
Symbiodinium spp. clade C	767		69,018		50		Shoguchi et al., 2018 [25]
Symbiodinium spp. clade A	705		65,832		43		Shoguchi et al., 2018 [25]
Symbiodinium tridacnidorum CCMP2592	1,103	85.71		651.3	51.01		González-Pech et al. 2021 [23]

^a^Information came from transcriptome and incomplete draft genome

### Dinoflagellate genomics landscape and incredible inter-species dissimilarities

To date, 28 dinoflagellate genomes have been sequenced (with two sequenced more than once), mostly from the family Symbiodiniaceae (Table 1). Sequenced taxa other than Symbiodiniaceae included *Amoebophrya* sp. ex *Karlodinium* *veneficum* [9], *Amoebophrya ceratii* [10, 11], *Amphidinium gibbosum* [12], *Hematodinium* [20], *Polarella glacialis* [21], and *Prorocentrum cordatum* (formerly *P. minimum*) [22]. These taxa cover a wide range of environmental conditions and different lifestyles, despite the obvious bias toward small end of the dinoflagellate genome size spectrum. Species from the Symbiodiniaceae are endosymbionts of tropical corals or their free-living counterparts. *Amoebophrya* species are intracellular parasites, infecting other dinoflagellates. *Amphidinium gibbosum* and *P. cordatum* form harmful algal blooms and *A. gibbosum* is toxigenic. *P. glacialis* thrives in the Antarctic and Arctic, can survive temperature shock to 15ºC [26] and DNA barcoding suggests that this and closely related species occur in the temperate estuary Long Island Sound [27].

Most of these genome assemblies were based on Illumina Hi-Seq technology and hence are largely fragmentary. However, using PacBio to generate long sequence reads and Hi-C to establish linkages between scaffolds, chromosome level assemblies have been achieved for part of the *F. kawagutii* genome (> 120Mbp; [19]) and the genomes of *Symbiodinium microadriaticum* [28] and *Breviolum minutum* [29].

The number of chromosomes varies vastly between species, ranging from several to more than 200 [30]. By microscopic count of DAPI-stained chromosomes and telomere end count (divided by 2), *F. kawagutii* has between 7 [31] and 11 chromosomes [18]. In sharp contrast, there are 94 chromosomes in *S. microadriaticum* [28] and 91 in *B. minutum* [29]. While the number for *S. microadriaticum* is comparable to a previous microscopic estimate (100; [32]), that for *B. minutum* exceeds five-fold the microscopic count of ~ 17 (Fig. 1 in [13]). This inter-species disparity is remarkable given that the genome size among these species differs by no more than 2-fold. Equally striking are their compensating differences in chromosome length. The chromosome length is markedly shorter in *S. microadriaticum* (16Kb to 16.6 Mb, median 6.6 Mb; [28]) and in *B. minutum* (median 6.7 Mb, longest ~ 11 Mb; [29]) than in *F. kawagutii* (maximum > 120 Mb; [19]). These data suggest an interesting evolutionary trend of chromosome number decrease and chromosome length increase from the basal *S. microadriaticum* to later-diverging *F. kawagutii* [33]. Based on the existing data, the small non-parasitic dinoflagellate genomes such as those of Symbiodiniaceae contain approximately 1–3 Gbp DNA distributed in 10–100 chromosomes of 100 − 10 Mbp each; the largest dinoflagellate genome contains approximately 250 Gbp DNA likely distributed in 200–1000 chromosomes of 1.25 Gbp-250 Mbp each.

**Fig. 2:**
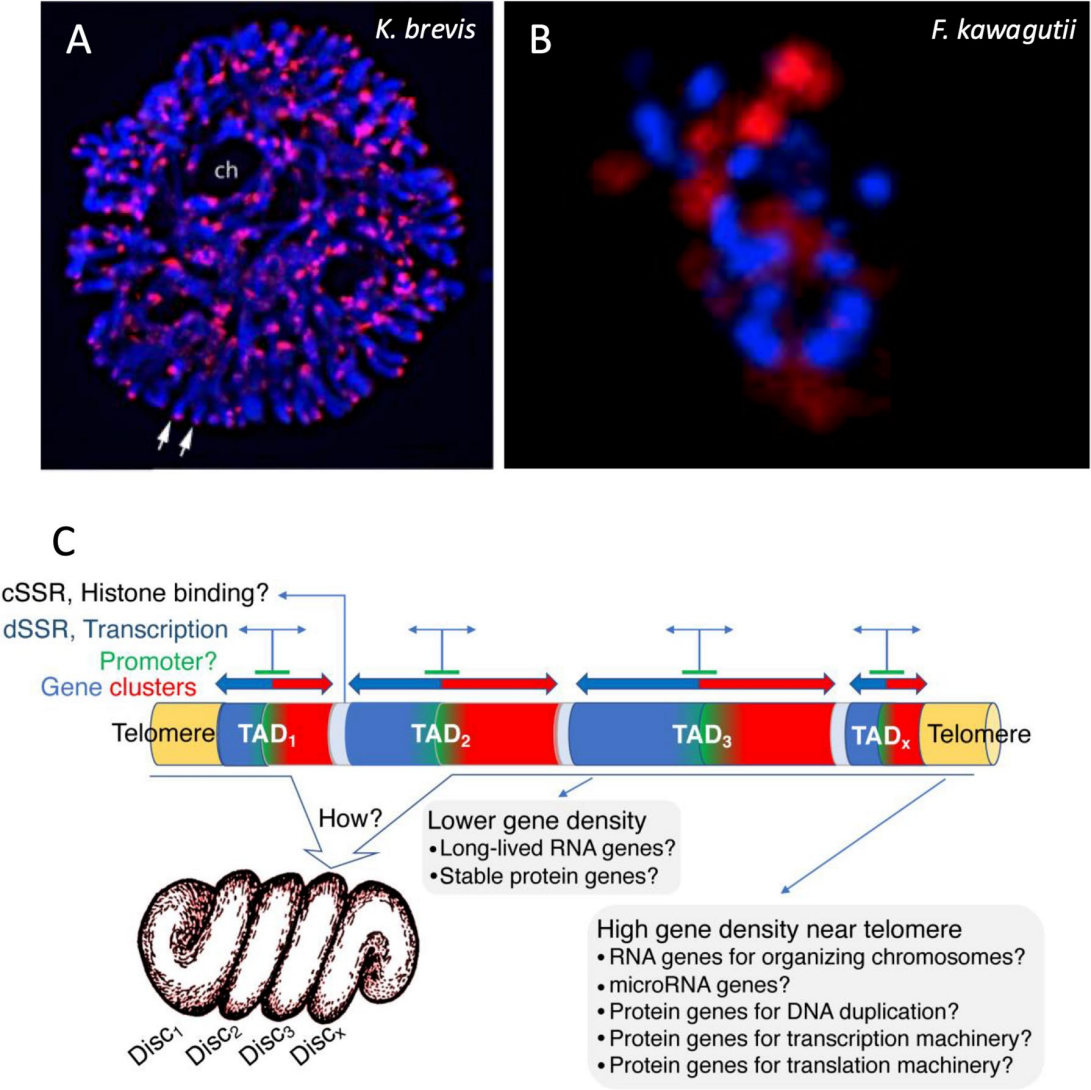
Distribution pattern of telomeres in a tertiary endosymbiosis dinoflagellate and schematic of TADs in dinoflagellate chromosomes and associated gene distribution and expression machinery. **A** Chromosomes of *Karenia brevis *stained with fluorescent in situ hybridization of telomere probe. **B** DNA DAPI stain (Blue) and telomere FISH (red) in *F. kawagutii*. **C** TAD organization highlighting gene distribution and gene expression machinery. Reproduced with permission from Cuadrado et al. 2019 [37] (**A**), Lin et al. 2015 [18] (**B**), and Lin et al. 2021 [33] (**C**)

Genome sequencing combined with fluorescent in situ hybridization (FISH; Fig. 2A, B) revealed that the telomere sequence in *F. kawagutii* consists of repeats of TTTAGGG, depicted as (TTTAGGG)_n_, which is identical to the typical plant telomere (Richards and Ausubel 1988), as is also true for other dinoflagellates [34, 35]. The length of the telomere tract was estimated at 25–80 Kbp in *Karenia papilionacea* and *Crypthecodinium cohnii* [35] but is unclear in other species. Using non-denaturing fluorescent in situ hybridization, Cuadrado et al. (2022) [36] revealed bipolar distribution of clustered multiple foci of telomeric DNA: one in the nucleolus organization area and the other peripherally along the convex side of the nucleus, suggesting that the telomeres change their positions during cell division.

Dinoflagellate genomes are highly divergent. This is reflected in the genomes of Symbiodiniaceae, among which a > 98% dissimilarity is consistently found by mapping reads from one genome to the assembly of the other genome [18, 23]. Whether this extreme divergence reflects rapid genome evolution in dinoflagellates in general or is due to severe isolation between Symbiodiniaceae species by their facultative symbiotic lifestyle remains to be investigated.

### Topologically associated domains (TAD)

Dinoflagellate genomes form topologically associated domains (TADs; Fig. 2C), a feature widely found in eukaryotic genomes [28, 29]. TAD is a genomic region where DNA sequences within the same TAD interact with each other more frequently than with sequences outside of it [38]. Between the two species that have been examined, *S. microadriaticum* has about 450 TADs with a median size of 1.8 Mb, whereas *B. minutum* contains 580 TADs with a median size of 5.7 Mb. These are markedly larger than mammalian TADs (~ 0.18 Mb) and bacterial CIDs (~ 0.17 Mb), and do not show interactions with more distant regions of the chromosome in the Hi-C maps.

In these two genome assemblies, genes are distributed along the chromosomes as an alternating series of unidirectional clusters [28, 29]. TADs show two types of strand switch regions (SSR) (Fig. 2C). One, where values of the DNA contact probability plot are closest to the diagonal axis in Hi-C interaction maps, corresponds to the end of transcription (a convergent strand switch region, cSSR). The other, where regions of the TADs show DNA interactions furthest from the diagonal corresponds to the initiation of transcription (a divergent SSR, dSSR). Roughly half the genes in the genome are found in groups of at least nine, all sharing a common direction. TAD boundaries are also associated with decreased GC content.

The dinoTAD structure suggests that transcriptional promoters might be found in the central region of the TADs where genes are oriented outward in both directions (Fig. 2C). These sites could represent histone attachment points, reconciling the conservation of histone sequences with their paucity [39].

The average interaction frequency between two regions of the chromosome decreases when the distance between them increases. The trend in *S. microadriaticum* [28] suggests that DNA in this species is organized as linear rods. Comparison to similar analyses with other genome organizations shows that although dinoflagellate nuclei presumably use viral nuclear protein (DVNP) and bacterium-originated histone-like proteins more than histones (due to their higher expression levels), the chromosome organization appears more like eukaryotic chromosomes, with the highest similarity to mitotic chromosomes in metaphase when the chromosomes are highly condensed [28, 33].

Marinov et al. (2021) [29] and Nand et al. (2021) [28] noted that when the transcription was blocked, the TADs disappeared, revealing transcription’s critical role in maintaining the TADs in dinoflagellate chromosomes. This aligns with earlier findings on RNA’s importance in chromosomal DNA structure [40], though recent data suggest transcription activity may be more crucial than RNA in stabilizing domain structure.

### Mechanisms of genome expansion: Duplication, (retro)transposition, and repetitive DNA

**Fig. 3:**
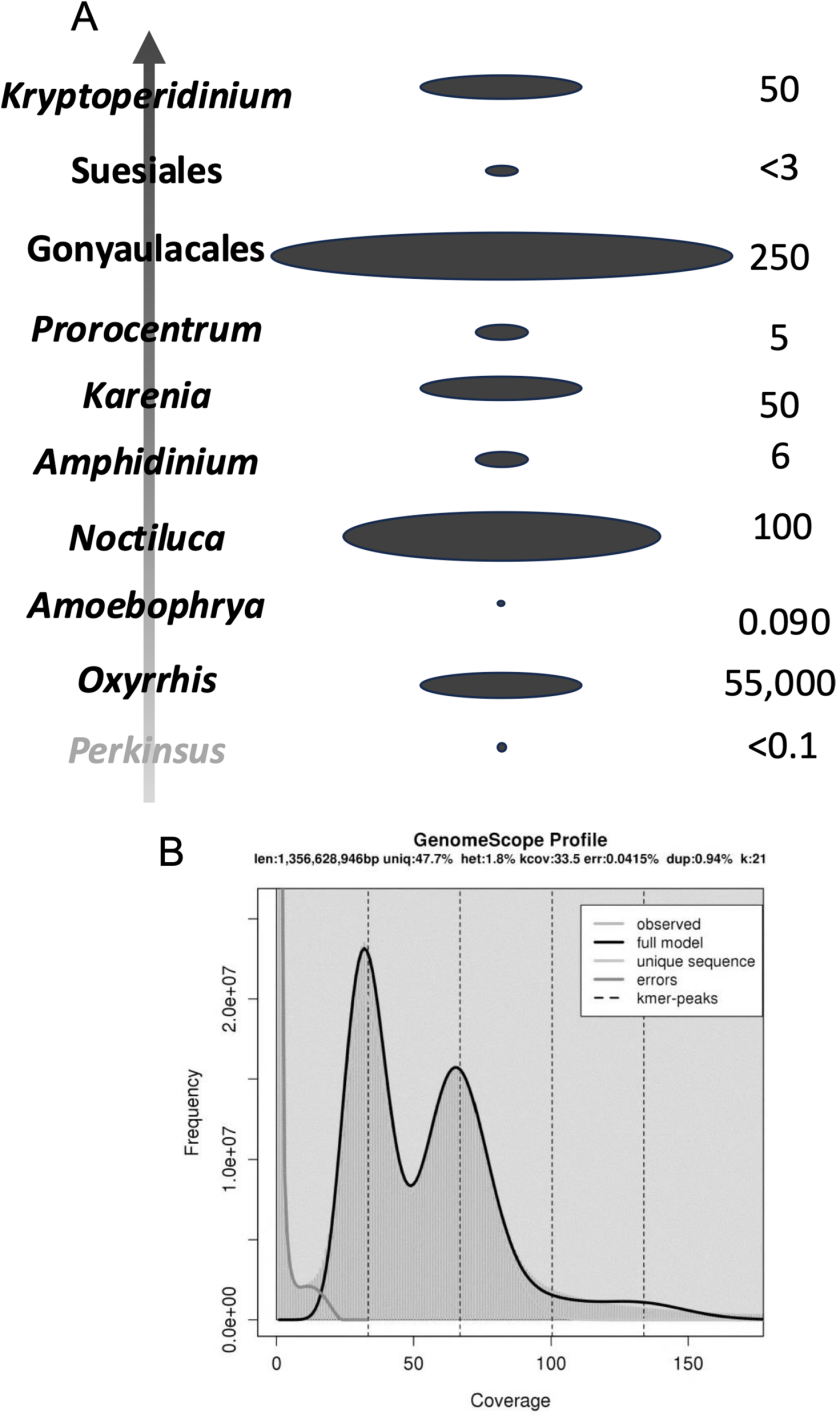
Evolutionary dynamics of dinoflagellate genome size showing no phylogenetic trend (**A**) and double peak in 21-mer profile of Polarella glacialis showing evidence of genome duplication (**B**). **A** Vertical arrow indicates phylogenetic trend from the dinoflagellate sister group perkinsids, basal dinoflagellate *Oxyrrhis*, to later diverging taxa. Size of oval in the middle illustrates genome size (not to scale) as depicted by numbers shown on the right. **B** Reproduced from Stephans et al. 2020 [21] under the terms of the Creative Commons CC BY license

Dinoflagellate genome sizes vary widely among species, from < 0.1 Gbp in the parasitic taxon *Amoebophrya*, < 3 Gbp for most coral symbionts in the family *Symbiodiniaceae*, to ~ 250 Gbp for free-living taxa [10, 41, 42]. There is no phylogenetic trend in the vast genome size variation, however (Fig. 3A). For example, the coral symbiotic lineage *Symbiodiniaceae* has the smallest genomes among documented dinoflagellates despite its later-diverging position in the phylogenetic tree [43]. The large genome sizes and wide range among dinoflagellates suggest multiple independent events of genome expansion in evolution. Alternatively, genome contraction might have occurred occasionally, independent of symbiosis lifestyle [17].

Whole genome duplication (WGD) is the most efficient way to achieve chromosome multiplication and genome expansion [44, 45]. The immense dinoflagellate genomes might have resulted from WGD. However, K-mer profile and scaffold synteny (proxy of chromosome synteny) analyses for *F. kawagutii* did not show any signs of WGD [18]. Nevertheless, the genome of *Polarella glacialis* (~ 3 Gbp) is about double the size of that in *Symbiodiniaceae* [21]. Its k-mer profile, based on sequencing reads, shows double peaks characteristic of diploid genomes [21], suggesting a genome duplication (Fig. 3B). “Diploid”-like chromosome pairs have been reported for *Pyrocystis lunula* as well [46]. There is a good chance that WGD is widespread in large dinoflagellate genomes. However, the original interpretation of these observations as diploid is not consistent with the traditional view that dinoflagellates are haploids.

Besides WGD, genome expansion can result from processes like the proliferation of mobile DNA elements, including transposons and retrotransposons. The role of transposable elements in remodeling genomes has been demonstrated in plants [47]. Indeed, there is evidence that transposons [10] and retrotransposons [48] play roles in dinoflagellate genome expansion. The role of retrotransposition is corroborated by the prevalent gene paralogs and DinoSL (complete or truncated) at the upstream region of genes, which account for > 20% of total genes [49], made possible by the large families of reverse transcriptase and integrase genes in the genomes [18, 28]. In contrast, transposition is rare in the small genome (< 0.1 Gbp) of the parasitic species *Amoebophrya ceratii* [10]. González-Pech et al. (2021) [23] compared genome features of *S. tridacnidorum* and *S. natans* and postulated that retrotransposition might be a major driving force for the two genomes to diverge.

Stephens et al. (2020) detected a substantial proportion of long terminal repeat (LTR) elements (~ 12%) in *P. glacialis* genomes [21]. Transposable elements (such as LTRs) can comprise up to 80% of plant genomes and are induced by genome shock and polyploidization, leading to genome restructuring [50]. The abundance of LTRs in *P. glacialis* and their role in genome restructuring may explain the difference in genome size between populations. In *S. microadriaticum*, heat stress-activated Ty1-copia-type LTR retrotransposons were detected, and recent expansion was documented [51], suggesting proliferation of retrotransposable elements for adaptation to hot habitats. The survival of retroposed genes may be promoted by their methylation [52], as DNA methylation is thought to silence and promote transposition [53]. Similar TE domestication by epigenetic regulation seems common in eukaryotes [54], and methylation of transposable elements is presumably a major driving force of genome expansion in all eukaryotes [55].

The abundance of repetitive DNA also contributes to genome size variation. Using FISH, Cuadrado et al. (2022) [36] revealed clusters of dense trinucleotide repeats in the bean-shaped nuclei of *Karenia selliformis* and *K. mikimotoi*. Larger dinoflagellate genomes tend to contain more repetitive elements [21]. For instance, repetitive elements account for 23.8% of the 0.1156-Gbp *Amoebophrya* A120 genome, 13.1% of the 0.1160-Gbp *Amoebophrya* A25 genome, 27.9% of the ~ 1.10-Gbp *Symbiodinium microadriaticum* genome, and 16% of the ~ 1.2-Gbp *Fugacium kawagutii* genome [14]. For *P. glacialis*, with genome sizes of ~ 3 Gbp, the level of repetitive elements increases to ~ 68% [21]. *Crypthecodinium cohnii* has a ~ 7 Gbp genome [56], with 55–60% composed of repetitive DNA [57]. A survey of the ~ 115 Gbp *Alexandrium ostenfeldii* genome indicated that repetitive DNA accounted for ~ 58% of the genome [58]. Overall, these results suggest that repetitive elements significantly contribute to genome size evolution in dinoflagellates.

### Genomic innovation by rampant horizontal gene transfer (HGT)

Horizontal gene transfer (HGT) might be a significant driver of gene innovation in dinoflagellates [59]. HGT is widespread in eukaryotes, accounting for 0.1-6.4% of total genes, depending on the species [60]. While the full extent of HGT in dinoflagellates awaits the sequencing of large-genome species, current data provide ample evidence. Using EST data, Norsenko and Bhattacharya (2007) [61] identified 16 proteins acquired through ancient HGTs in the common ancestor of the genera *Karenia* and *Karlodinium*, and one protein from a more recent HGT. Eight of these proteins appear to have resulted from independent HGTs in several eukaryotic lineages, with many involved in energy metabolism. Most of these genes were transferred directly from bacteria, but some originated from another chromalveolate that had acquired them from bacteria. Some HGT-derived genes serve essential functions in dinoflagellates and warrant further discussion.

Genome sequencing for both the Antarctic (CCMP1383) and the Arctic (CCMP2088) strains of *Polarella glacialis*, compared with other dinoflagellate genomes, revealed putative bacteria-to-dinoflagellate HGT for ice-binding domain-containing proteins [21]. Several bacterium-originated genes were identified in the plastid genomes of *Pyrocystis lunula* and *Ceratium horridum*, including FtsY, Rpl28, Rpl33, Yef16, and Ycf24 [62]. *F. kawagutii* genome analysis also revealed 56 putative bacterium-originated HGT genes (0.12% of total gene models), of which 22 are flanked by eukaryotic genes on at least one side [18]. These included the erythromycin esterase superfamily gene, radical SAM protein, and Phosphoadenosine phosphosulphate (PAPS) reductase. Erythromycin esterases (IPR007815) disrupt erythromycin by hydrolyzing the macrolactone ring, thereby conferring antibiotic resistance (InterPro). Radical SAM proteins (IPR007197) catalyze diverse reactions, such as unusual methylations, isomerisation, sulfur insertion, ring formation, anaerobic oxidation, and protein radical formation in biosynthesis and biodegradation pathways of DNA precursors, vitamins, cofactors, antibiotics, and herbicides (InterPro). PAPS reductase (IPR002500) is part of the adenine nucleotide alpha hydrolases superfamily, which includes N-type ATP PPases and ATP sulfurylases; this enzyme uses thioredoxin as an electron donor for the reduction of PAPS to phosphor-adenosine-phosphate (PAP) (InterPro).

#### Form II Rubisco

One of the most notable bacterium-originated HGT genes in dinoflagellates is Form II Rubisco (Rubisco II). Rubisco II exists only in peridinin-containing dinoflagellates and anaerobic bacteria [63–65]. Possibly because of its anaerobic bacterial origin, Rubisco II is more sensitive to O_2_ and less efficient at CO_2_ fixation than Form I Rubisco, which is prevalent in oxygenic photosynthetic organisms.

#### Proton pump rhodopsin (PPR)

PPR (also known as proteorhodopsin) is another remarkable bacterium-originated HGT genes in dinoflagellates. Initially found in prokaryotes from a metagenomic library [66], PPR is now known to be widespread in bacteria [67] and various protists lineages, including dinoflagellates [68] and diatoms [69]. This transmembrane protein, carrying an *all-trans* retinal chromophore, functions as a photoreceptor harvesting light energy.

Dinoflagellate PPR expression increases in response to dim light, starvation, and phosphorus (P) deficiency [70–72]. In *Prorocentrum shikokuense*, PPR was upregulated 40-fold during a P-depleted bloom outbreak (Zhang et al. 2019). Studies consistently indicate PPR’s potential role in providing energy during nutrient stress.

PPRs in dinoflagellates localize on plasma or endosomal membranes. GFP-fused PPR genes from *P. shikokuense* and *Alexandrium catenella* (Group I) showed cell membrane localization in HEK293 cells [73]. *Oxyrrhis marina* possesses both proton pump and sensory rhodopsin [70, 74], with unique distribution on endosomal, plasma, and vacuolar membranes [74–76].

Some dinoflagellate genomes encode variants of microbial rhodopsin genes, including a giant ion channel formed by a rhodopsin gene fused with a bestrophin gene [77].

#### Dinoflagellate viral nuclear protein (DVNP)

Frequent viral infection [78–80] and colonization in a non-lytic fashion [81] provides copious opportunities for invasion into dinoflagellate genomes. The recent detection of a CRISPR-Cas enzyme in *Symbiodinium pilosom* [82] suggests that dinoflagellates might have evolved an anti-viral defense mechanism similar to that in bacteria. Frequent viral infection creates opportunities for HGT. The acquisition of DVNP [83], which will be further discussed in the next section, is hitherto the most prominent case of virus-to-dinoflagellate HGTs. The probable functional replacement of DVNP for histones in structuring chromatin [84] testifies for the remarkable impact of HGT on the biology of the host.

### Loss of nucleosome and gains of nuclear proteins: A nuclear drama like no other

Chromosome structure is influenced by DNA binding proteins, cations, and RNA. Mg^2+^ and Ca^2+^ are important in DNA configuration [85–87]. RNA may also be involved in DNA packaging [40, 87]. Abundant RNA binding proteins were detected in the extracts of purified chromatin by high throughput proteomic analysis in *Lingulodinium polyedra* [88].

**Fig. 4:**
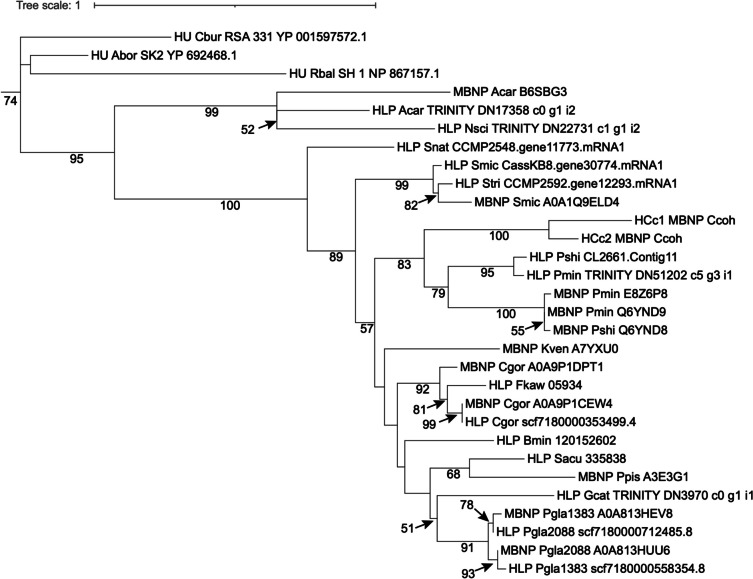
Phylogenetic tree of major basic nuclear protein (MBNP), histone-like protein (HLP), and bacterial DNA binding protein (HU)-like protein (Hcc). The tree is rooted with bacterial DNA binding protein (HU). The lack of monophyletic separation indicates that these three proteins belong to the same protein family under different names. Species name abbreviations: Abor, Alcanivorax borkumensis; Acar, Amphidinium carterae; Bmin, Breviolum minutum; Cbur, Coxiella burnetiid; Ccoh, Crypthecodinium cohnii; Cgor, Cladocopium goreaui; Fkaw, Fugacium kawagutii; Gcat, Gymnodinium catenatum; Kven, Karlodinium veneficum; Nsci, Noctiluca scintillans; Pgla, Polarella glacialis; Pmin, Prorocentrum minimum; Ppis, Pfiesteria piscicida; Pshi, Prorocentrum shikokuence; Rbal, Rhodopirellula baltica; Sacu, Scrippsiella acuminata; Smic, Symbiodinium microadriacticum; Snat, Symbiodinium natans; Stri, Symbiodinium tridacnidorum

Dinoflagellate nuclei have a 10-fold lower nuclear protein to DNA ratio (1:10) than typical eukaryotes (1:1) and lack nucleosome (Rizzo 1991) despite presence of core histones [39, 68]. Recent studies suggest low amounts of nucleosomes may occur [89, 90]. Dinoflagellates have acquired alternative proteins through HGT. One of these proteins is the major basic nuclear protein (MBNP), the coding gene of which is one of the most highly expressed genes in dinoflagellates [2]. Two related proteins have abundantly reported: histone-like proteins (HLP) and bacterial DNA binding protein (HU)-like protein (Hcc) [91, 92]. Phylogenetic analysis (Fig. 4) suggests that MBNP, HLP, and HCc belong to the same protein family, thus warranting a unifying name. MBNP appears to be the most suitable to represent this gene family.

Another acquired protein is dinoflagellate/Viral nucleoproteins (DVNP). DVNP acquisition coincided with nucleosome disappearance early in dinoflagellate evolution [83]. Heterologous expression of a DVNP in *Saccharomyces cerevisiae* showed that DVNP expression inhibited growth, diminished histones, disrupted nucleosomes, and impaired transcription, detrimental effects that were relieved by histone depletion [93]. These results suggest that histone abandonment might be due to the ‘colonization’ of the ‘invading’ viral protein.

Dinoflagellates appear to have lost histone H1 [94]. HCc exhibits about 10% similarity to H1, implying that HCc might be a functional replacement of H1 [95]. The similarity could cause misidentification of HCs as H1, which can explain reports of H1 presence [89]. Stringent in silico studies suggest that H1 is absent in dinoflagellates [94]. Experimental evidence is required to nail the issue.

### Gene structure: Unidirectionality and intron dynamics

Unlike typical eukaryotes, dinoflagellate genes are dominantly unidirectionally arranged in the genome, forming co-oriented clusters [13, 18, 22]. This characteristic is widespread in dinoflagellates, including basal *Amoebophrya* spp [11]. Such organization might facilitate transcription in unison and in foci on certain areas of the TADs [28, 29, 33].

Contrary to previous belief, dinoflagellate genes are not intron-depleted. Even in streamlined parasitic *Amoebophrya ceratii* genomes, 67-71% of genes contain introns, with 64–99% in Symbiodiniaceae species (Table 1). The ~ 6.4 Gbp *(A) gibbosum* genome has fewer introns per gene but longer total intronic regions compared to smaller dinoflagellate genomes. Average intron lengths vary: 335–345 bp in *Amoebophrya* spp., 505, 517, and 893 bp for *S. microadriaticum*, *(B) minutum*, and *F. kawagutii*, respectively [11]. Notably, a 26,372 bp intron was detected in *(C) cohnii* [96].

Intron-exon boundaries in dinoflagellates also differ from typical eukaryotes [11]. Besides the canonical GT-AG boundary, dinoflagellates use GCIGA-AG (25–74% in Symbiodiniaceae; <3% in *Amoebophrya*), and other boundaries (up to 67% in *Amoebophrya*). Interestingly, 99.98% of introns in *Amoebophrya* strain AT5 have canonical GT-AG splice sites, while over 60% in strains A25 and A120 have non-canonical splice sites.

Intron creation may be driven by introners (specialized transposable elements). Analysis of five dinoflagellate genomes revealed: (1) evidence of historical intron creation, (2) recently active introners in four of five species, (3) dynamic intron gain and loss, with higher gain rates than most eukaryotes, and (4) In *P. glacialis*, 12,253 introns from recent introner insertion, with 15 distinct families accounting for at least 100 introns each [97].

Data to date show that dinoflagellates use standard codons. However, exceptions have been reported, mostly in the stop codon. In *Amoebophrya* sp. ex *Karlodinium veneficum*, as demonstrated in dynein heavy chain genes, all three stop codons are inferred to code for amino acids [9]. UAA and UAG appear to encode glutamate whereas UGA either serves as the stop codon or encode tryptophan.

### High gene content, complex organization, and abundant “dark” genes

#### High gene content

A predictive correlation between protein-coding genes and genome size was developed in Hou and Lin (2009) [98]: log (gene number) = LN (-46.200 + 22.217*log[genome size]), where genome size is in Kbp. This empirical model predicts 38,188 to 87,688 protein-coding genes for dinoflagellate genomes of ~ 2 to 250 Gbp. However, recent genome data show higher gene numbers than predicted in most cases (Table 2), suggesting dinoflagellate genome expansion disproportionately favors gene increase compared to other eukaryotes.

**Table 2:** Comparison of identified gene number with predicted gene number based on Hou and Lin (2009) [98]

Species	Genome size (Mbp)	Detected gene no.	Model-predicted gene no.	% deviation^a^
Parasitic				
* Amoebophyra ceratii* AT5.2	120	19,925	15,834	26%
* Amoebophyra* sp. A25	116	28,091	15,656	79%
* Amoebophyra* sp. A120	115.5	26,441	15,633	69%
* Hematodinium* sp.^a^	4800	29,510	42,412	-30%
*Symbiotic*				
* Breviolum minutum*	616	41,925	25,830	62%
* Cladocopium goreaui*	1,200	45,322	30,710	48%
* Durusdinium trenchii*	670	30,000	26,419	14%
* Fugacium kawagutii*	935	45,192	28,828	57%
* Symbiodinium microadriacticum* CCMP2467	808	49,109	27,758	77%
Free-living				
* Effrenium voratum* RCC1521	1,200	32,108	30,710	5%
* Effrenium voratum* CCMP3420	1,300	39,878	31,328	27%
* Effrenium voratum* CCMP421	1,100	32,615	30,046	9%
* Polarella glacialis* CCMP1383	2,980	58,232	38,147	53%
* Prorocentrum cordatum*	4,750	85,849	42,316	103%

^a^(detected-modeled)/modeled x 100

#### Shared and unique genes and gene families

**Fig. 5:**
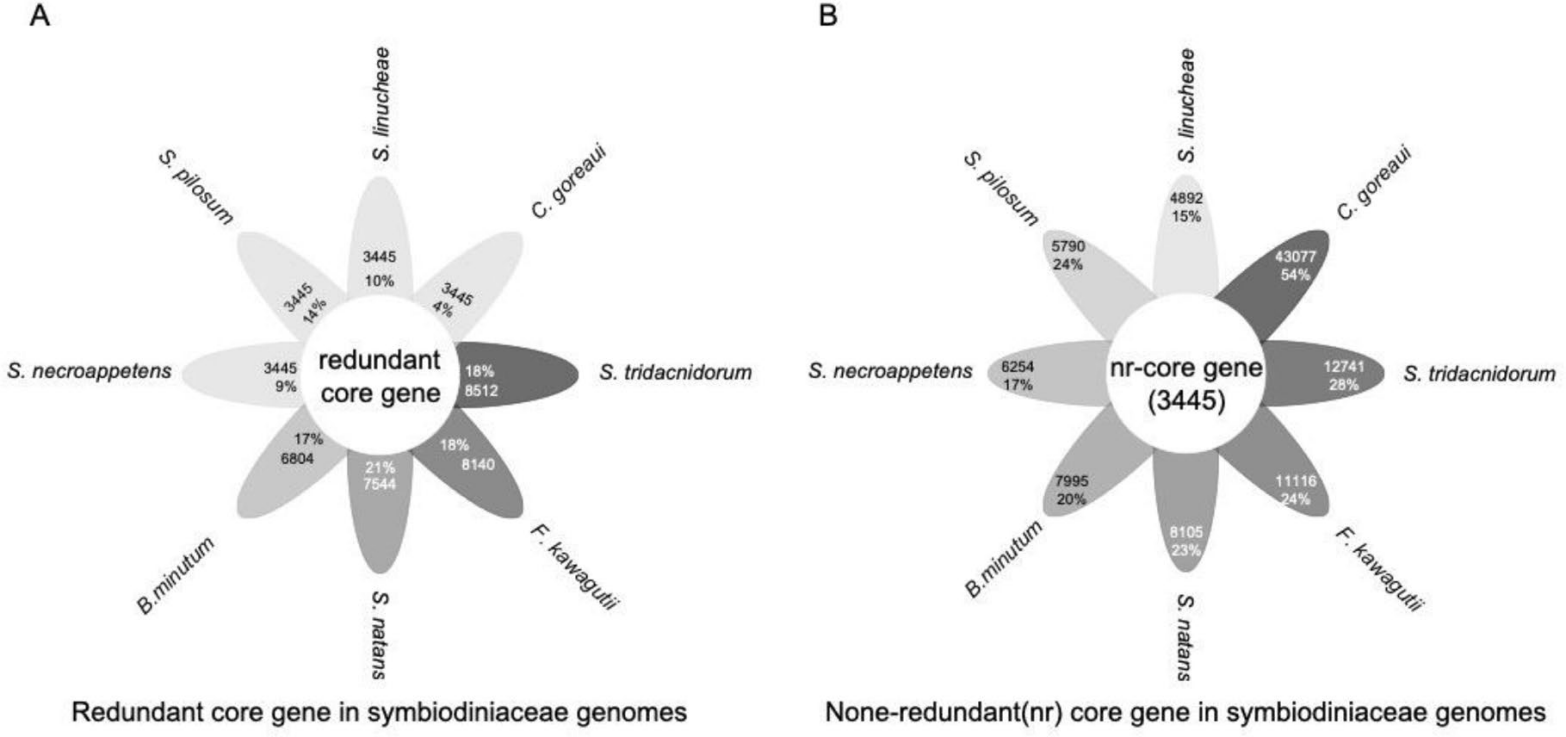
Shared and unique genes in Symbiodiniaceae genomes. **A** Core genes (3,445 after de-redundancy) in proportion of total genes in each species. **B** Unique genes in each species. Darker colors indicate a larger number of genes

Current genome sequencing efforts for dinoflagellates have biased toward species with smaller genomes, precluding identification of phylum-wide common genes. Nevertheless, comparative genomic analyses still provide interesting insights into differential dynamics and diversification of dinoflagellate genomes. Analysis of 15 Suessiales genomes found 555,682 predicted protein sequences, with 42,539 forming gene families [23]. Only 2,500 (5.88%) have homologs in all 15 isolates, indicating most gene families are lineage-specific. Within Symbiodiniaceae, 3,445 unigenes are shared by eight species, representing core genes enriched in 219 KEGG pathways (Fig. 5).

#### Highly expressed and most abundant genes

Previous transcriptomics data showed highly expressed genes including MBNP, glyceraldehyde-3-phosphate dehydrogenase (GAPDH), peridinin-Chl *a*-binding protein (PCP), heat shock protein (HSP) 90, HSP70, HSP40, elongation factor EF-1α, S-adenosyl-L-methionine (SAM) synthetase, A-adenosyl-homocysteine hydrolase (SAHase), and calmodulin [2]. Now with more data, the highly expressed genes exhibit more inter-specific difference (Table 3).

**Table 3:** Ten most highly expressed genes in dinofalgellates (shading indicates grouping by gene name)

Species	Ranking												Reference
	1	2	3	4	5	6	7	8	9	10	11	12	
Non-Symbiodiniaceae	**MBNP**	**GAPDH**	**PCP**	**HSP90**	**HSP70**	**HSP40**	**EF-1α**	**SAMS**	**SAHase**	**CaM**			Lin et al. 2011 [2]
*Cladocopium goreaui*	**PCP**	**atpE**	unk^1,2^	**CaM**	**MBNP**	**FAM135A**	unk^3^	**PSMG1**	**psbD**	unk^4^	unk^5^	**RPL28e**	Huang and Lin unpubl
*Durusdinium trenchii*	**PEX16**	**EFG**	**GAPDH**	**UBE**	**USP7**	**PCP**	unk^8^	**psbJ**	**EF-1α**	**petJ**	unk^9^	**TUBB**	Huang and Lin unpubl
*Effrenium voratum*	**PCP**	**psbA**	**petJ**	unk^10^	**petF**	unk^11–12^	**LHCP**	unk^13^	**RPL**	unk^14–16^	**HSP40**	**Ub**	Li T et al. 2021 [99]
*Fugacium kawagutti*	**psbA**	**petF**	**NRT2.5**	**PCP**	unk^18–19^	**psbV**	**rbcL**	**PGK**	**psbC**	**psaA**	**LHCP**		Li et al. 2020 [19]
*Karenia mikimotoi*	**HLP**	**MBNP**	**LHCP9**	**LHCP12**	**Ub**	unk^20^	**LHCP4**	**LHCP**	**LHCP8**	unk^21^	**actin**	unk^22^	Lin et al. 2022 [100]
*Karenia mikimotoi*	unk^23^	**psbA**	unk^24^	**SAG-L**	**MBNP**	**HLP**	**psbD**	**psaB**	**atpH**	**petB**	**rbcL**	**psbV**	Lin et al. 2022 [100]
*Prorocentrum shikokuense*	**SAG-L**	unk^25–26^	**LSU-rRNA**	unk^27–28^	**SKP1**	**MBNP2**	**psbA**	**psbE**	**psbD**	**PCP**	unk^29–30^	**petB**	Zhang et al. 2019 [101]
*Prorocentrum shikokuense*	unk^25^	**SRm300**	**psbA**	unk^31–32^	**psbD**	unk^33–34^	**MBNP2**	**RPS15A**	**RPS30**	unk^35–37^	**RPL36**	**RHO**	Yu et al. 2020 [102]
*Prorocentrum shikokuense*	**MBNP2**	**SRm300**	**PCP**	unk^41^	**FCP**	unk^42^	**atpE**	unk^34^	**RHO**	**MBNP**	**HSP40**	**petB**	Li H et al. 2021 [103]

Pfam analysis for *A. gibbosum* genome revealed Leucine-rich repeat (LRR), Ankyrin, Tetratricopeptide (TPR), and Pentatricopeptide repeat (PPR) domains as the most abundant domains in this species [12].

#### Tandem gene arrays and polyprotein expression

Many dinoflagellate genes occur in tandem arrays, likely a result of unequal crossing over in meiosis, replication slippage during DNA replication, retroposition, DNA transposons, and gene conversion [104]. Some of these tandem-arrayed genes are transcribed multi-gene transcripts, which is then *trans*-spliced, with the addition of a 22-nt highly conserved spliced leader (DinoSL; [105]), to generate individual mature mRNA with DinoSL at the 5’ end and poly A tail at the 3’ end. Others are expressed as polyprotein: each array is transcribed as a poly-gene transcript and translated into a poly-protein peptide, which is subsequently cleaved into individual proteins. Genes expressed as polyproteins documented to date in dinoflagellates include the chlorophyll *a*-*c* binding protein [106], luciferase [107], as well as Rubisco II [104, 108]. The Rubisco II polyprotein contains a signal peptide at the N-terminus of the first protein unit guiding the polyprotein’s transport to the chloroplast. At the chloroplast, the polyprotein is cleaved into individual Rubisco with a transit peptide at the N terminus to facilitate entrance into the plastid, where the transit peptide is cleaved [2, 104]. The polyprotein gene arrays were also found from the recent genome sequencing [22]. While tandem repeats are common in dinoflagellates, polycistronic expression appears rare, as manifesting in *L. polyedrum* [109].

Polyprotein genes such as Rubisco II in dinoflagellates likely have resulted from retroposition-based gene duplication, because the gene cluster contains only one regulatory system, without stop codons between individual proteins in the tandem array, characteristic of retroposed genes [104]. Polyprotein expression is generally less common in eukaryotes than viruses, but TY1 retrotransposon in yeast and certain plant storage proteins such as in seeds, and prohormones in mammals are expressed as polyproteins. Interestingly, viruses express both polycistronic and polyprotein genes; some viruses use polycistronic mRNAs, while others use polyproteins. Further research is required to address the mechanism underlying the formation of tandem repeats and polyprotein genes in dinoflagellates.

#### “Dark” genes

About 50% or more of predicted genes in dinoflagellate genomes lack functional annotation. This applies to both small (*Amoebophrya*) and large (*P. cordatum*, *A. gibbosum*) genomes [11, 12, 22]. Some dark genes are common across taxa, but most are species-specific [23, 110]. These may have crucial functions, as shown in a bloom metatranscriptomic study [101].

### Gene expression and regulation: Roles of cold shock proteins, microrna, and alternative splicing

Dinoflagellates are generally known to have limited transcriptional regulation (5–27% of genes; for review see Lin 2011 [2]). However, significant transcriptional responses to nutrient changes have been reported [111, 112]. For example, alkaline phosphatase gene expression and enzyme activity increase under phosphate limitation [112–115]. Nitrogen deficiency elicits significant upregulation of acquisition genes and downregulation of nitrogen associated protein gene (NAP50) [116]. In addition, Rubisco, antenna protein, and some cell cycle protein genes show strong diel dynamics of transcript abundance [72, 108]. Earlier studies indicated that several percentage of genes exhibited circadian rhythms of transcript abundance at 2- or more fold variations [117]. These findings suggest that dinoflagellates may have more dynamic transcriptional regulation than previously thought, particularly in response to environmental changes and daily cycles.

#### General gene expression

General transcriptional machinery in eukaryotes includes RNA polymerase II transcription initiation factors (TFII), TFIIA, TFIIB, TFIID, TFIIE, and TFIIH. These factors assemble on promoter DNA alongside polymerase II to form a multiprotein-DNA complex crucial for precise initiation of basal-level transcription [118]. Additionally, transcriptional activators may bind to the enhancer region and coactivators may mediate interactions between enhancer-bound activators and promoter-bound gene-specific transcription factors (TFs), regulating transcription responses to environmental cues [119].

Of the five TFII genes, only one TFIID and three TFIIH genes were identified in the *F. kawagutii* genome [18, 118]. Dinoflagellates lack the typical eukaryotic TATA box (consensus sequence TATAAA) that interacts with TATA-binding protein (TBP), but instead use TTTT interacting with a TBP-like protein (TBL), as previously proposed and confirmed by genome data [18, 120].

#### Cold shock domain (CSD)

CSD-containing proteins (CSPs) were initially identified in *Escherichia coli* during cold shock stress and are now known to occur in major groups of organisms. In prokaryotes, CSPs act mainly as RNA chaperones and can regulate transcription by binding to the gyrA promoter. In eukaryotes, CSPs usually contain additional domains and play roles in cold stress response, nutrient limitation, and growth. In plants, CSPs are essential for freezing tolerance and regulate translation during cold stress, seed germination, and flowering [121].

CSPs are ubiquitous in dinoflagellates. In *L. polyedrum*, CSP genes were the only gene family with higher transcriptional levels than in other protists [109], suggesting their significant role. Some LpCSPs contain only the CSD domain while others have additional domains, but neither showed cold induction [122]. In *F. kawagutii*, at least eight of 121 identified transcription factors (TFs) contain CSD (Skav204535, Skav204536, Skav217190, Skav218283, Skv218284, Skv223430, Fkav224338, Skv231215), suggesting that CSPs may be the functional domains of TFs in dinoflagellates.

CSPs were identified in *Scrippsiella acuminata*, showing increased expression in resting cysts [123]. *Prorocentrum cordatum* has two CSD genes that respond significantly to low temperatures [124]. Dinoflagellate CSPs contain a ZF domain and a glycine-rich motif besides the conserved CSD, with varying ZF and GR order between species. This organization resembles bacterial and plant CSPs, suggesting possible bacterial origin through HGT.

**Fig. 6:**
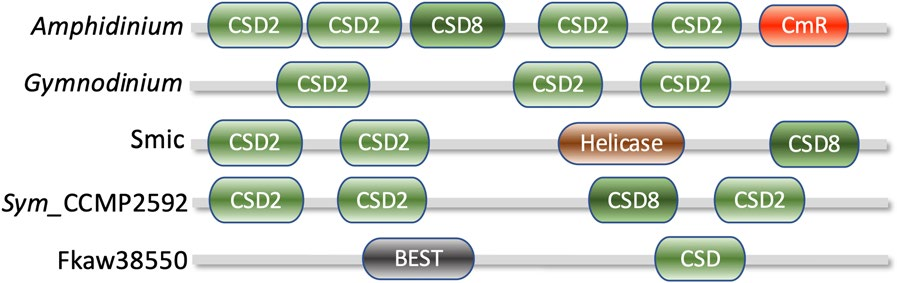
Organization of cold shock domain (CSD) in various proteins in dinoflagellates. *Amphidinium, A. massarti, *data source MMETSP0689; CSD2, domain PS51857; CSD8, domain SM00357; CmR, Calcium regulated mRNA-binding domain (IPR052069); *Gymnodinium*, *G. catenatum*, data source MMETSP0784-20121206; Smic, *Symbiodinium microadriaticum*, data source CAE7767176.1 dbp2, Helicase = helicase superfamily ATP-binding domain; Sym_CCMP2592, *Symbiodinium* strain CCMP2592; Fkaw38550, *Fugacium kawagutii*, BEST = bestrophin

DNA and RNA binding assays showed dinoflagellate CSD proteins prefer binding RNA over DNA, and single-stranded over double-stranded DNA, without sequence specificity [118]. This suggests they are unlikely to act as transcription factors but may be involved in DNA unwinding. Some CSPs contain calcium-regulated mRNA-binding or helicase ATP-binding domains in certain species (Fig. 6).

#### MicroRNA (miRNA)

The scarce and weak transcriptional regulation in dinoflagellates suggests other mechanisms must exist. MicroRNA repertoires have been documented in the transcriptomes of *Symbiodinium microadriaticum* [125] and *Prorocentrum shikokuence* [102, 114] and the genome of *F. kawagutii* [18].

Functional microRNA machinery is evidenced by effective RNA silencing (Zhang and Lin 2019). *A. gibbosum* genome analysis suggests microRNA involvement in regulating responses to nitrogen and phosphate starvation [12].

**Fig. 7:**
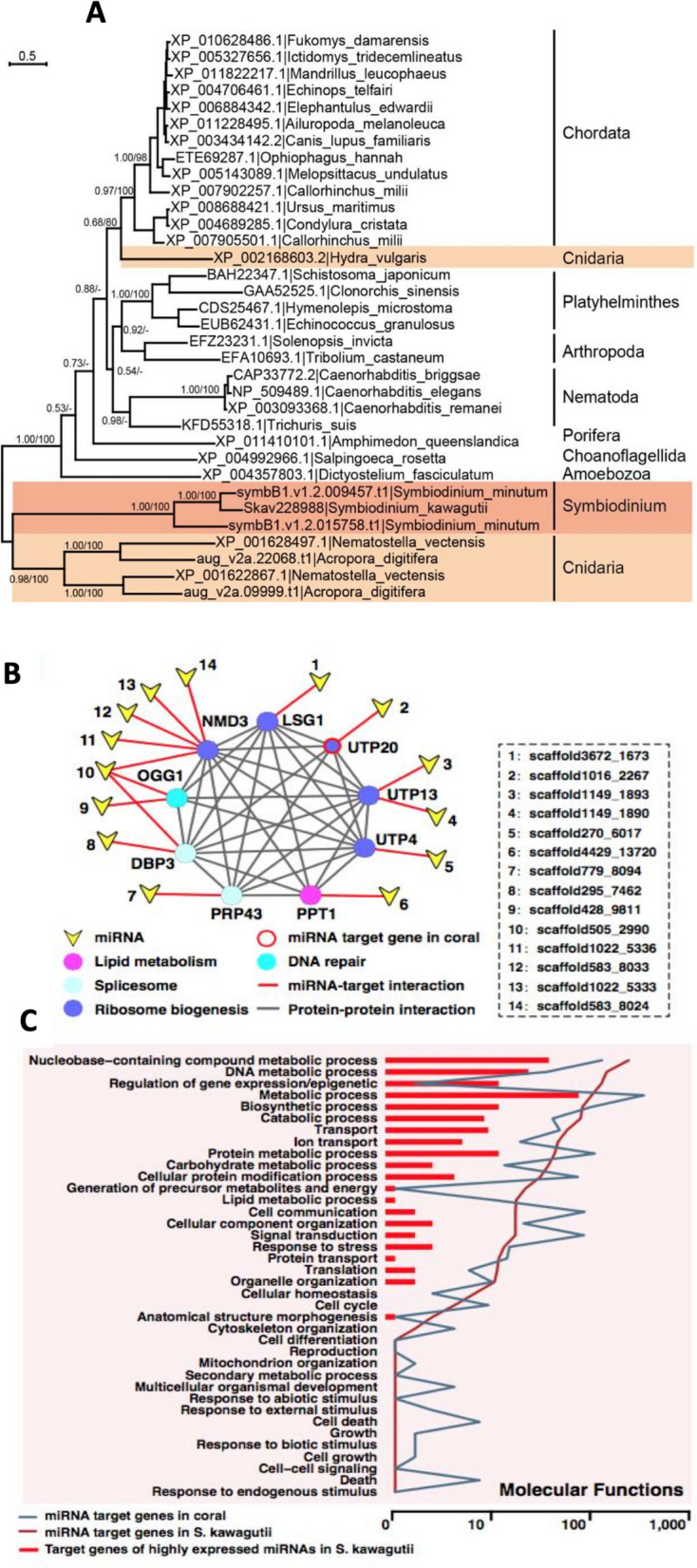
Evidence that Symbiodiniaceae miRNA may interact with coral host to modulate host gene expression. **A** Phylogenetic tree of double-stranded RNA channel (SID-1). The close relationship between the homologs in *Symbiodinium* and the cnidarians suggests horizontal gene transfer between the two lineages. **B** Protein interaction network and metabolic pathways predicted to be impacted by miRNA. **C** GO categorization of predicted miRNA target genes in both *F. kawagutii *and coral

In silico analyses of *F. kawagutii* miRNAs indicated potential influence on coral host gene expression [18]. The genome contains a putative RNA transporter homologous to Systemic RNA Interference Deficiency–1 (SID-1) in nematodes and mammals. Close relationship between Symbiodiniaceae and cnidarian homologs suggests possible HGT (Fig. 7A). Potential target genes of symbiodiniacean miRNA were identified in coral hosts, showing broad interactive protein network (Fig. 7B) and GO terms (Fig. 7C).

These findings suggest Symbiodiniaceae microRNA may cross over into the invertebrate host and modulate gene expression, potentially impacting symbiosis mechanisms like nutrient uptake and photosynthate translocation. While not experimentally demonstrated, this is consistent with findings in other symbiotic and pathogenic systems. *Vibrio fischeri* produces noncoding small RNA that controls host gene expression in squid light organs [126, 127]. Pathogen-to-host miRNA transfer silences host immunity genes in plants [128].

#### Alternative splicing

Genome analyses have detected alternative splicing in dinoflagellates. In *A. gibbosum*, a high number of alternative splicing events were identified, potentially responsible for gene diversification, particularly in secondary metabolite biosynthesis pathways [12]. The majority (77%) of the alternative splicing events occur by skipping exons, and the rest uses alternative 3′splice sites (A3SS) or alternative 5′splice sites (A5SS) (6.8% and 11.3%, respectively). These events mainly impact ion transport, nucleic acid metabolism, and RNA metabolic process. Notably, polyketide synthase (PKS) genes have alternatively spliced isoforms and the genes are expressed as polycistronic. Alternative splicing is also suspected in *F. kawagutii* and other dinoflagellates [19, 110]. However, the extent and function of alternative splicing in dinoflagellates remain largely unknown.

#### Post-transcriptional regulation

**Fig. 8:**
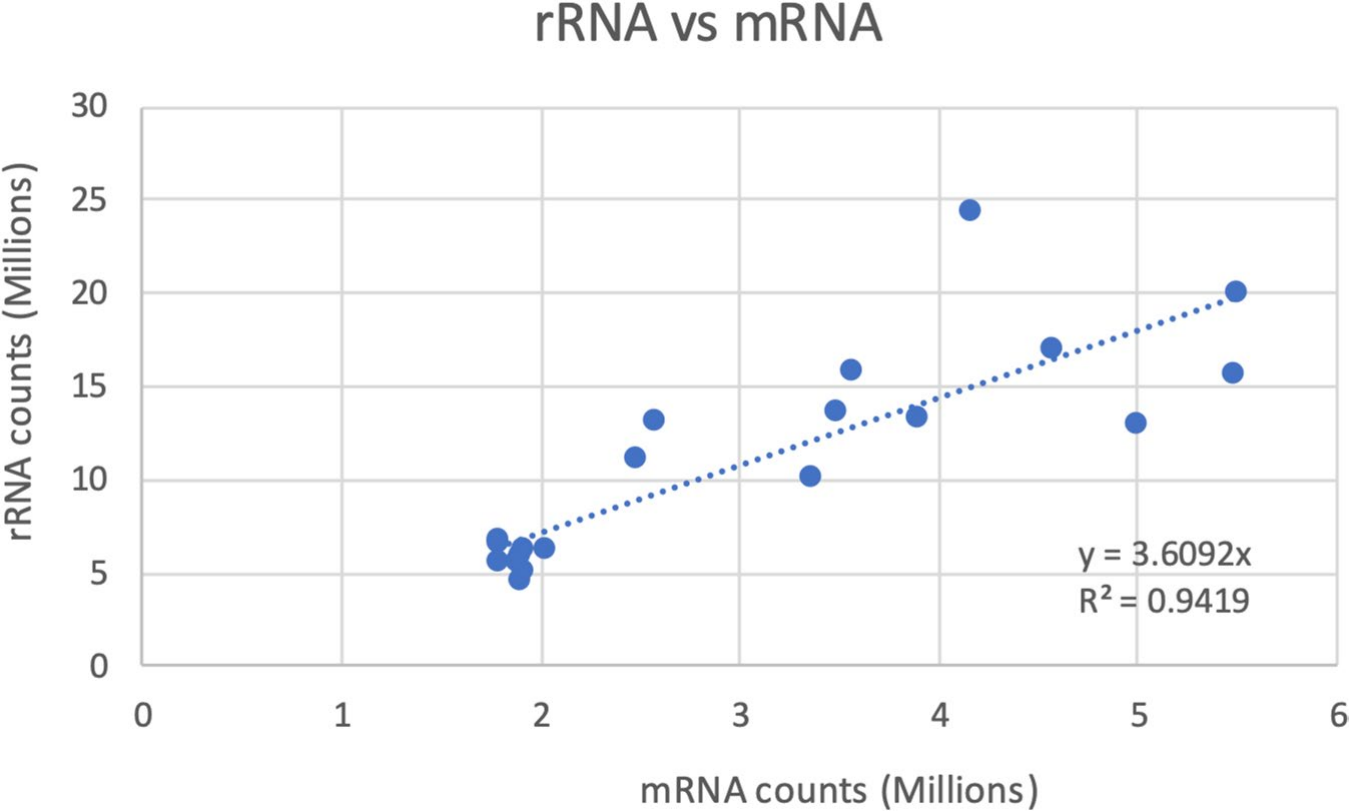
Correlation between ribosomal rRNA and ribosome-bound mRNA abundances in *Lingulodinium polyedra*. Data from Bowazolo et al. (2022) [129]

Ribosome-protected mRNA profiling revealed pulses of synchronous translation of gene clusters throughout the diel cycle [129], indicating circadian or light/dark modulation of gene expression. Pearson correlation analysis of rRNA count versus mRNA count shows that on average, translation of an mRNA requires 3.6 ribosomes (Fig. 8). Deviations from this may indicate above or below average translational efficiency, with exceptionally high translation activity occurring at LD20 (four hours before light-on).

A study of 11 core dinoflagellate transcriptomes revealed diversity in the eIF4E family [130]. Each species contained 8–15 (average 11) different eIF4E family members, phylogenetically segregated into three clades. Dinoflagellate eIF4E-1 likely contains at least one translational initiation factor. eIF4E-2 lacks mRNA cap-binding residues and may have a non-essential regulatory role. eIF4E-3 genes share attributes with metazoan Class I eIF4E. The three clades were further divided into nine subclades, six containing members from all eleven species, suggesting ancestral duplication.

### Powerhouse of RNA editing

RNA editing is a gene modification process where single or multiple nucleotides in a gene are altered during or after transcription. This can be identified by comparing the nucleotide sequence of a gene with its corresponding mRNA. RNA editing occurs in mRNA, tRNA, and rRNA, involving mitochondrial, plastid, and nuclear genes [131].

In dinoflagellates, RNA editing was first documented in mitochondrial protein-coding gene transcripts, showing extensive and mechanistic diversity [132]. Mitochondrial mRNA editing has increased in the percentage of impacted nucleotides throughout dinoflagellate evolution [27]. Most nucleotide changes are synonymous and do not affect the encoded proteins but tend to increase the GC content of the impacted genes. This may enhance translation efficiency since dinoflagellate mitochondria import cytoplasmic tRNA for translation, which may not function well with AT-rich mitochondrial genes [27]. This explanation, however, does not apply to rRNA editing.

The field of RNA editing in dinoflagellates has advanced significantly in the last decade. mRNA editing of mitochondrial protein-coding genes has been extended to basal, parasitic dinoflagellates such as *Amoebophrya* [9]. Furthermore, editing was also found in chloroplast-encoded transcripts, including mRNA and rRNA [133, 134]. For coral symbiotic Symbiodiniaceae, RNA editing was detected in *in-hospite* populations [135]. Interestingly, mitochondrial gene transcripts are more frequently edited than plastid genes [135]. RNA editing occurs not only in typical peridinin-containing dinoflagellate plastids but also in Chl *b*-containing plastids in *Lepidodinium* spp. and fucoxanthin-containing tertiary plastids of haptophyte origin in *Karlodinium veneficum* [136–138]. Whether plastids in the Dinotom group, such as *Durinskia kwazulunatalensis* with diatom-origin plastids, also undergo RNA editing remains unclear. This, along with the accelerated evolution of plastid protein-encoding genes in peridinin- and fucoxanthin-containing species, indicates a significant host effect [139].

Furthermore, mRNA editing of nucleus-encoded genes has also been detected. For instance, one of the saxitoxin biosynthesis genes, sxt4, was found to undergo editing in *Alexandrium pacificum* [140]. Additionally, using high-throughput sequencing, mRNA editing has been detected in nuclear genes. Up to 10,486 and 69,953 putative RNA editing sites were identified in the nuclear genomes of the coral symbiont *D. trenchii* CCMP2556 and the free-living bloom-forming species *P. cordatum* CCMP1329, respectively [22]. These events included all 12 possible types of RNA edits, with more transitions than transversions and a dominance of A-to-T transversion in noncoding regions, many of which were condition-specific. Furthermore, A-to-T editing within untranslated regions appears to be associated with the upregulation of edited genes under heat stress [15]. If experimentally verified, the high number of RNA editing events suggests that RNA editing may be a more influential molecular mechanism regulating gene expression in dinoflagellates than previously thought.

### New insights on dinoflagellate chloroplasts

Nucleomorphs, relic nuclei of eukaryotic endosymbionts, have been found in some dinoflagellate chloroplasts. Previously known only in the phylum Cryptophyta [141], a recent study discovered nucleomorphs in two newly described dinoflagellate species with green chloroplasts [142]. These chloroplasts, like those in *Lepidodinium* spp., originate from prasinophytes, although *Lepidodinium* spp. do not contain nucleomorphs.

Typical dinoflagellate chloroplast genomes are composed of minicircles, each containing one or two genes (occasionally none), totaling 16–17 genes per plastid genome [143]. In contrast, plastid genomes in *Durinskia baltica* and *Kryptoperidinium foliaceum* are large macrocircles, similar to typical algal plastids, and more similar to those of diatoms [144]. Chloroplast proteins in dinoflagellates evolve faster than in other algal lineages, indicating a strong host effect on plastid evolution [139].

**Fig. 9:**
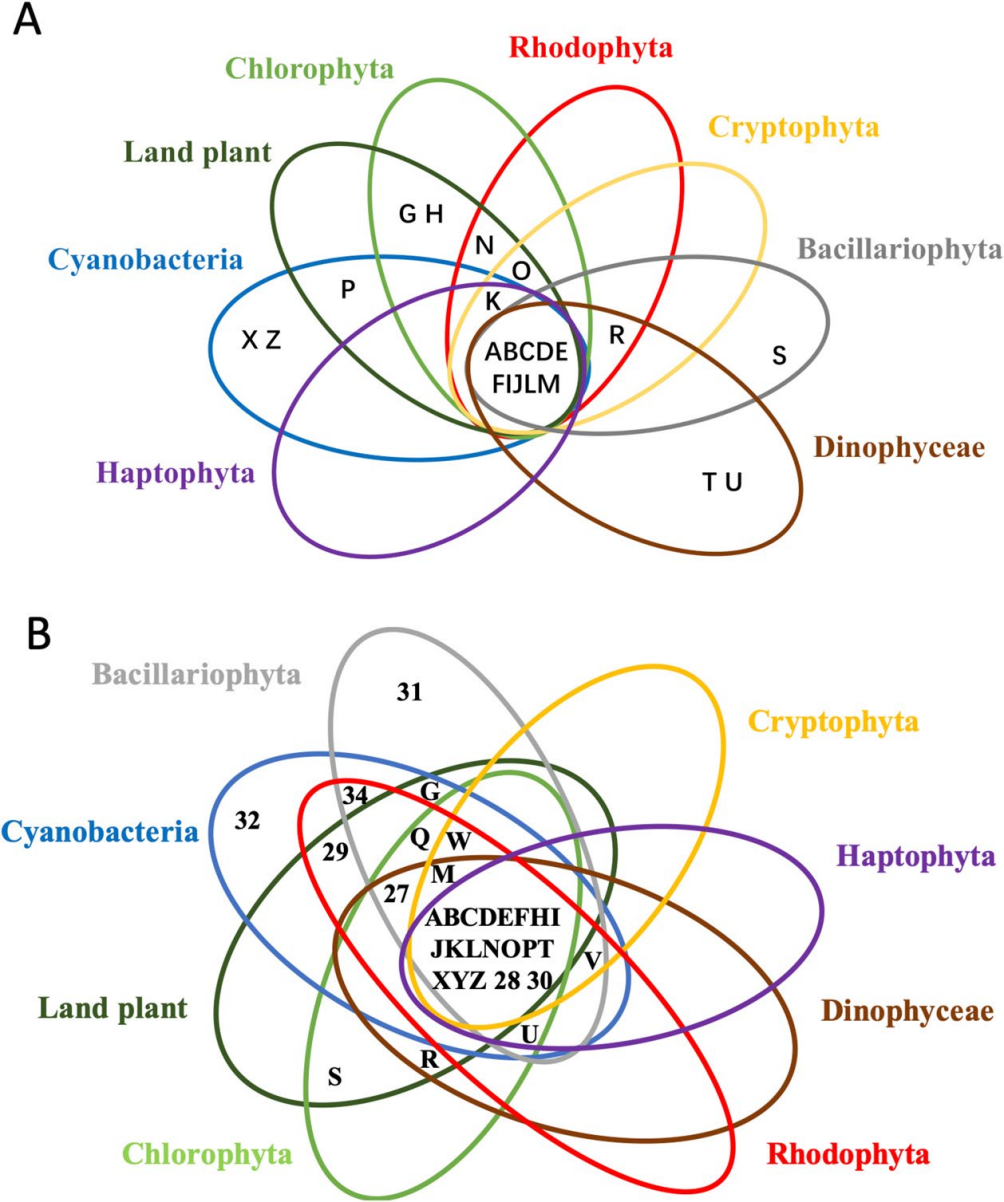
Comparison of dinoflagellate photosystem (PS) components with counterparts in other algal lineages. **A** PSI. Reproduced from Lin et al. 2024 [149]. **B** PSII. Information source: Fromme et al. 2001 [150], Pi et al. 2018 [151], Qin et al. 2019 [152], Gisriel et al. 2022 [153], Su et al. 2019, 2022 [154, 155], Zhao et al. 2023 [156], Li et al. 2024 [82]

Dinoflagellates have conserved photosystem cores (PSI and PSII) but varied light-harvesting complexes (LHCI and LHCII). Dinoflagellates have 12 subunits in PSI, ten of which are shared by all photosynthetic organisms, and 24 in PSII, 20 of which are shared by all photosynthetic organisms (Fig. 9). Dinoflagellate LHCs consist of a thylakoid intrinsic Chl *a*-Chl *c2*-peridinin-protein complex (acpPC) and a water-soluble peridinin-Chl *a*-protein (PCP) complex [145, 146]. Peridinin is the major accessory pigment (carotenoid) unique to dinoflagellates, which is used to define typical dinoflagellates. PCP exists in two forms, differing in absorption and fluorescence properties [147, 148].

Single-particle electron microscopy of PSI-LHCI supercomplexes in *Breviolum minutum* revealed 25 LHCs, including LHCF and LHCR families, and indicated a role in photoprotection via nonphotochemical quenching [157]. Recent cryo-EM studies identified novel PSI subunits (PsaT and PsaU) and suggested enhanced electron transport and energy quenching efficiency [82, 158].

Photochemical experiments have shown high quenching efficiency in dinoflagellates, such as symbiodiniacean species. Under stress conditions, *Cladopodium* cells activate a “super-quenching” mechanism, transferring excess excitation energy to PSI and converting it into heat, potentially triggering symbiont expulsion or bleaching [159].

### Adaptation to symbiotic lifestyle

**Fig. 10:**
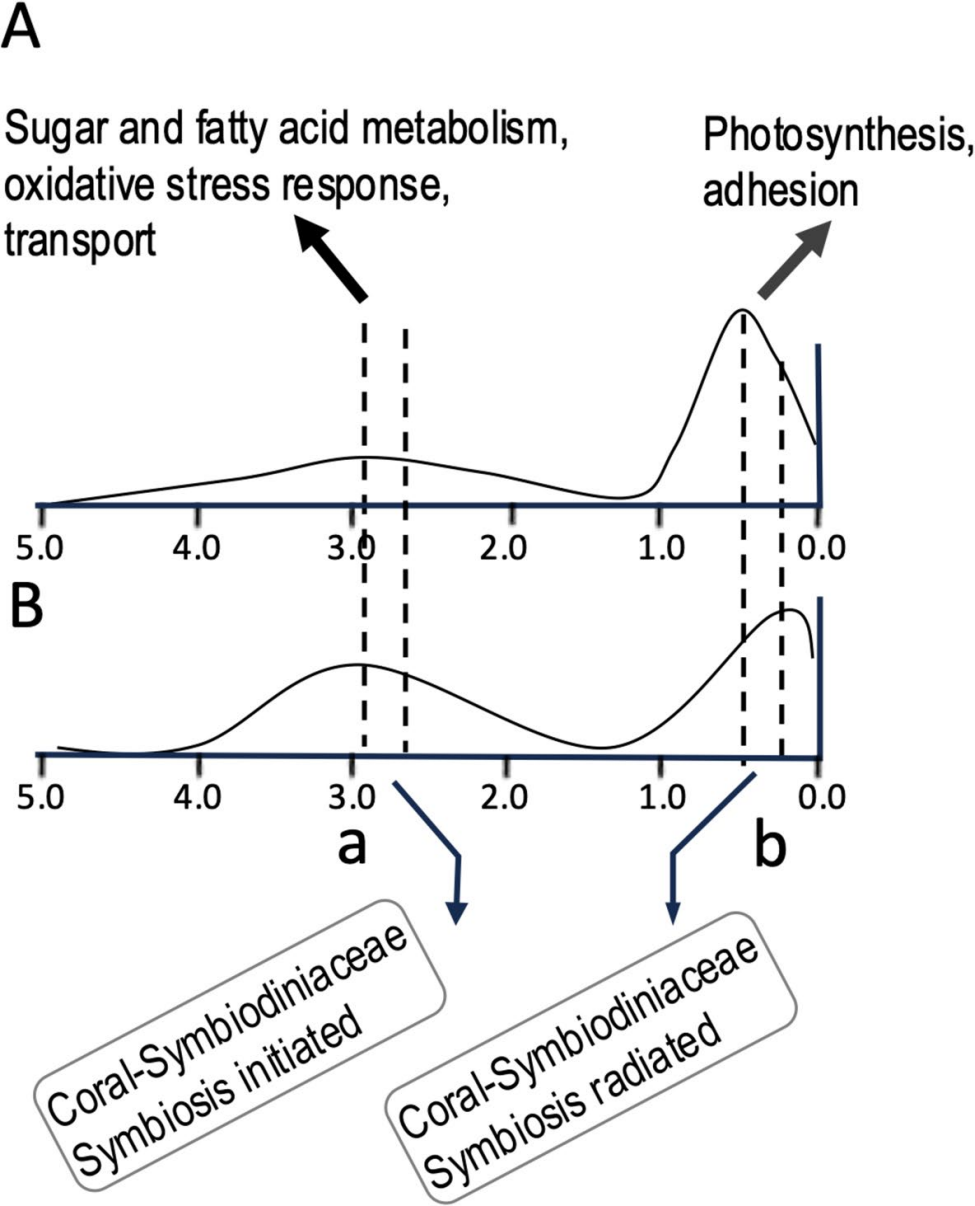
Two episodes of retroposition discovered in two Symbiodiniacea genomes coincident with two major periods of symbiosis evolution. A) *Fugacium kawagutii. *B) *Breviolum minutum*. a, first episode led to expansion of sugar and fatty acid metabolism, oxidative stress response, and transport. b, second episode that promoted photosynthesis and adhesion. Based on Song et al. 2017 [49]

Comparative genomic analyses reveal that genes involved in nutrient uptake, photosynthate transport, stress response, and infection are present and expanded in symbiodiniacean dinoflagellates [18, 24]. In the genomes of *B. minutum* and *F. kawagutii*, genes important for mutualism, such as those related to sugar and fatty acid metabolism, oxidative stress, transport, photosynthesis, and adhesion, have undergone duplication during two major retroposition events (Fig. 10; [49]). Comparison of symbiodiniacean genomes with those of other algae, the land plant *Arabidopsis thaliana*, and the parasite malaria *Plasmodium falciparum* identified genes unique to *Plasmodium* and Symbiodiniaceae, such as those involved in cell recognition (e.g. merozoite surface protein 1) and genes with unknown functions (e.g. uncharacterized protein PF11_0213, protein dpy-19 homolog 1) [18]. In *Plasmodium*, merozoite surface proteins are crucial for red blood cell invasion (Beeson et al. 2016).

The *F. kawagutii* genome encodes 16 putative circumsporozoite protein (CSP)-coding genes and a CSP-like gene with a forkhead domain, potentially regulating vesicle-mediated transport and CSP secretion. In the chlorophyte symbiont *Symbiochlorun hainanensis*, a CSP-like protein is upregulated by acidification and heat stress [160]. Protein secretion via the T2S system, encoded by at least 12 genes, is a major virulence mechanism in bacterial infections and may participate in bacteria-eukaryote mutualism [161–163].

UV protection is crucial in coral-Symbiodiniaceae mutualism. Mycosporine-like amino acids (MAAs) prevent UV damage, initially believed to be synthesized only by the basal lineage (clade A) of Symbiodiniaceae [164]. Genome analyses confirmed the presence of MAA biosynthesis genes in *S. tridacnidorum* and absence in later-diverging lineages like *F. kawagutii* and *Cladocopium* [18, 25], although an improvement of the genomes later revealed partial MAA biosynthesis genes in *F. kawagutii* [19]. Furthermore, the MAA gene cluster was found to be present in *D. trenchii*, in addition to *Symbiodinium*, but absent in *B. minutum*, consistent with the original notion of ancestral synthesis ability with secondary loss [16].

Insights into mutualism were gained from green *Noctiluca scintillans*, which harbors prasinophyte *Protoeuglena noctilucae* as endosymbionts. This mixotrophic dinoflagellate forms massive blooms in the Arabian Sea, feeding on phytoplankton and producing ammonium for symbiont photosynthesis [165, 166]. During blooms, symbiont photosynthesis is active, but cell division is suppressed, suggesting the host promotes symbiont photosynthesis while inhibiting symbiont proliferation to maximize photosynthate translocation [166].

### Adaptation to parasitic lifestyle

*Amoebophrya* species are intracellular parasites of marine dinoflagellates, radiolarians, ciliates, and other *Amoebophrya* strains. The genomes of *Amoebophrya ceratii* strain AT5.2 and *Amoebophrya* sp. strains A25 and A120 are significantly streamlined, with sizes (~ 120 Mbp) much smaller than other dinoflagellates (~ 1-250 Gbp). They have few transposable elements, short introns and intergenic regions, and a limited number of gene families, encoding 19,925, 28,091, and 26,441 genes, respectively [10, 11]. These genomes are gene-rich, with 26%, 27%, and 80% more genes than predicted for AT5.2, A25, and A120, respectively, comparable to some other dinoflagellates (7-89% above model prediction; Table 2).

Most genes in all three species (56%, 63.7%, and 59%) lack functional annotation in public databases, suggesting some may be specific to parasitism. The intron density (about 1.3, 1.47, and 1.42 introns per kb of coding sequence in AT5.2, A25, and A120, respectively) is typical of dinoflagellates, but the introns are mostly non-canonical, including repeated introns with highly variable splicing motifs. The canonical GT-AG splice sites account for only 34.02% and 30.41% in strains A25 and A120, respectively, with the majority (> 65%) being various other boundaries. In these strains, 11-30% of the non-canonical introns contain 8–20 nt inverted repeat motifs and 3–5 nt direct repeat motifs, spreading similarly to transposable elements (TEs).

High gene block synteny (> 49%) but low gene sequence similarity (< 50%) was observed among the *Amoebophrya* strains, suggesting strong selection pressure for protein changes due to parasitic interaction with different hosts. All three strains exhibit organelle reduction, including loss of the plastid and potential loss of mitochondrial genome and functions, indicating adaptive evolution. This is consistent with findings in *Hematodinium*, another parasitic dinoflagellate, which infects shellfish and other organisms [18].

Transcriptome sequencing of *Hematodinium* revealed 29,510 unique ORFs with no evidence of nucleus-encoded plastid-targeted protein genes or plastid gene homologs. The same BLAST workflow revealed 94 putative mitochondrial proteins. Genes involved in fatty acid, lysine, and tetrapyrrole synthesis lack organelle-targeting signal peptides, suggesting these pathways occur in the cytosol. Interestingly, the type I fatty acid synthase (FAS) responsible for cytosolic FA synthesis appears to have taken the form of plastid type II FAS by fusing multiple protein genes [20].

### Highlight of newly found genes and physiologies

The rapidly growing dinoflagellate genomic data constitute a rich resource for exploring unique physiologies or metabolic features of this unique group of protists. The many functional unknown genes predicted from their genomes will also serve as a target of future studies. A few of these genes, which are highly interesting but have not received adequate attention, deserve some highlight here. They are involved in acquisition and storage of nutrients, defense (toxin production), response to stress, and life cycle.

#### Use of Cyanate as a Nitrogen Nutrient

*Alexandrium catenella* ( formerly *A. fundyense*) actively expresses cyanate lyase (cyanase) during toxic bloom outbreaks. Zhuang et al. (2015) observed cyanase expression in a Northport Harbor, Long Island Sound bloom, indicating nitrogen stress due to rapid population growth [116]. Metatranscriptomic data showed downregulation of nitrogen-associated proteins (NAPs) and active expression of urea and nickel transporters (nickel is a cofactor of urease) and urease. This suggests *A. catenella* utilizes all nutrient acquisition mechanisms, including cyanate metabolism. Metagenomics data further revealed that cyanase is globally prevalent in plankton, underscoring the role of cyanate metabolism in the nitrogen biogeochemical cycle [167].

#### Storage of Nitrogen as Uric Acid or Polyguanine

Dinoflagellates have long been thought to store nitrogen in uric acid crystals. NanoSIMS and transmission electron microscopy studies confirmed that Symbiodiniaceae species store nitrogen as uric acid crystals following sudden environmental nitrogen increases [168]. Pulses of ammonium, nitrate, or aspartic acid promoted uric acid crystal accumulation, which formed quickly and were remobilized within 24 h. Genome analysis of *Fugacium kawagutii* found genes promoting uric acid biosynthesis, such as xanthin dehydrogenase/oxidase [18].

Recent studies, however, showed that dinoflagellates also synthesize guanine polymers. Jantschke et al. (2019) [169] and Mojzes et al. (2020) [170] observed guanine crystals in *Calciodinellum operosum* and *Amphidinium carterae*, suggesting their function in light scattering into chloroplasts. In *A. carterae*, guanine supported growth as the sole nitrogen source for several generations, suggesting guanine crystals as nitrogen storage may be functionally equivalent to *cyanophycin* in cyanobacteria.

#### Phagotrophy Gene Repertoire

Phagotrophy, a mode of nutrition involving particle ingestion through endocytosis, is common among dinoflagellates. This process includes four major steps: endocytosis, phagosome formation, fusion of phagosomes with lysosomes, and autophagy [171, 172]. Phagotrophy is an ancient feature found in various organisms, including heterotrophic and photosynthetic protists [173–175].

Dinoflagellates, comprising 50% heterotrophic species, exhibit mixotrophy with phagotrophic capabilities [176, 177]. The molecular mechanisms of phagotrophy in dinoflagellates are still being uncovered. Genes involved in autophagy and endocytosis, such as ATG3, ATG8, ATG12, PIP5K, PLD, AP2 adaptor complex, dynamin, epsin, Eps15, EH domain-containing protein 1, VPS4, VPS35, VPS45, Rab8, and Rab11, have been documented in laboratory cultures and natural assemblages [101–103, 178]. In a natural bloom of *Prorocentrum shikokuense*, genes involved in endocytosis, phagosome, peroxisome, and lysosome were actively expressed, particularly at night, suggesting increased feeding activity during nighttime [102].

These findings highlight the complexity and diversity of nutrient acquisition and storage mechanisms in dinoflagellates, emphasizing their adaptability and ecological significance.

#### Polyketide Synthase (PKS)/Non-ribosomal Peptide Synthetase (NRPS) Enzyme System and Secondary Metabolites

PKS/NRPS enzymes are crucial for producing specialized metabolites, including toxins, in dinoflagellates [179]. PKSs consist of an acyl-transferase (AT) domain, an acyl-carrier protein (ACP), and a ketosynthase (KS) domain and are classified into Type I, II, and III based on their organization [180]. Type I PKS has all catalytic domains on a single polypeptide, which is used in a processive fashion for chain elongation, as in animals and fungi. Type II PKS is a multiprotein complexe where each catalytic domain is found on a separate polypeptide. Type III PKSs, functioning iteratively similarly to Type II, are known from the plant chalcone/stilbene synthases (CHS/STS) producing compounds such as naringenin chalcone. NRPSs synthesize non-ribosomal peptides and include adenylation (A-domain), thiolation (T-domain), and condensation (C-domain) domains [181]. These enzymes can work together to produce hybrid natural products [179]. PKS and NRPS enzymes are found in toxin-producing dinoflagellates like *Karenia brevis*,* Gambierdiscus* spp., and *Amphidinium gibbosum* [12, 182–185] and in the symbiotic lineage Symbiodiniaceae [24, 186]. Dinoflagellates are unique in possessing both canonical Type I PKS and an unusual Type I sequence single-domain PKS (e.g. KS) [187, 188] and significantly expanded the PKS family relative to other protists [189]. A study scanning 47 dinoflagellate transcriptomes for modular synthase domains and their co-occurrence with thiolation domains revealed widespread presence, but in particularly high abundance in Gymnodiniales, of the thiolation domains [190]. Some of these occur alongside tetratricopeptide repeats (especially hexa- and hepta-repeats), which are unique to dinoflagellates.

#### Cyclophilins

Cyclophilins are a family of proteins known for binding cyclosporin A (CsA), an immunosuppressant drug in humans [191]. These proteins possess peptidyl-prolyl cis-trans isomerase activity, essential for correct protein folding. Cyclophilins have diverse functions, including protein folding, signaling, transcriptional regulation, cell cycle control, and stress response [192]. In algae, cyclophilins have been reported in *Chlamydomonas* [193] and *Ulva* [194], where they are implicated in immunity and stress response. For dinoflagellates, cyclophilin genes were detected over two decades ago (Lin and Zhang 2010), but their functions remain unclear. Recent studies indicate cyclophilins in *P. cordatum* are induced by copper and polychlorinated biphenyl exposure, suggesting a role in stress response [195]. Cyclophilin B in *Margalefidinium polykrikoides* is implicated in environmental stress responses [196].

#### Macrophage Migration Inhibitory Factors (MIF)

MIFs are cytokines and chemokines essential for the vertebrate immune system, mediating innate immune responses [197]. These proteins are conserved across the tree of life, serving various cellular functions [198]. In free-living organisms, MIFs activate immune responses, promote immune cell proliferation, and inhibit p53-mediated apoptosis [199]. Parasitic species produce multiple MIF proteins to modulate host immune responses, facilitating infection [200–203]. A homolog of MIF (PmMIF) was identified in the cyanobacterium *Prochlorococcus marinus*, showing high structural homology with mammalian MIFs but lacking the Cys-X-X-Cys motif necessary for oxidoreductase activity [204].

**Fig. 11:**
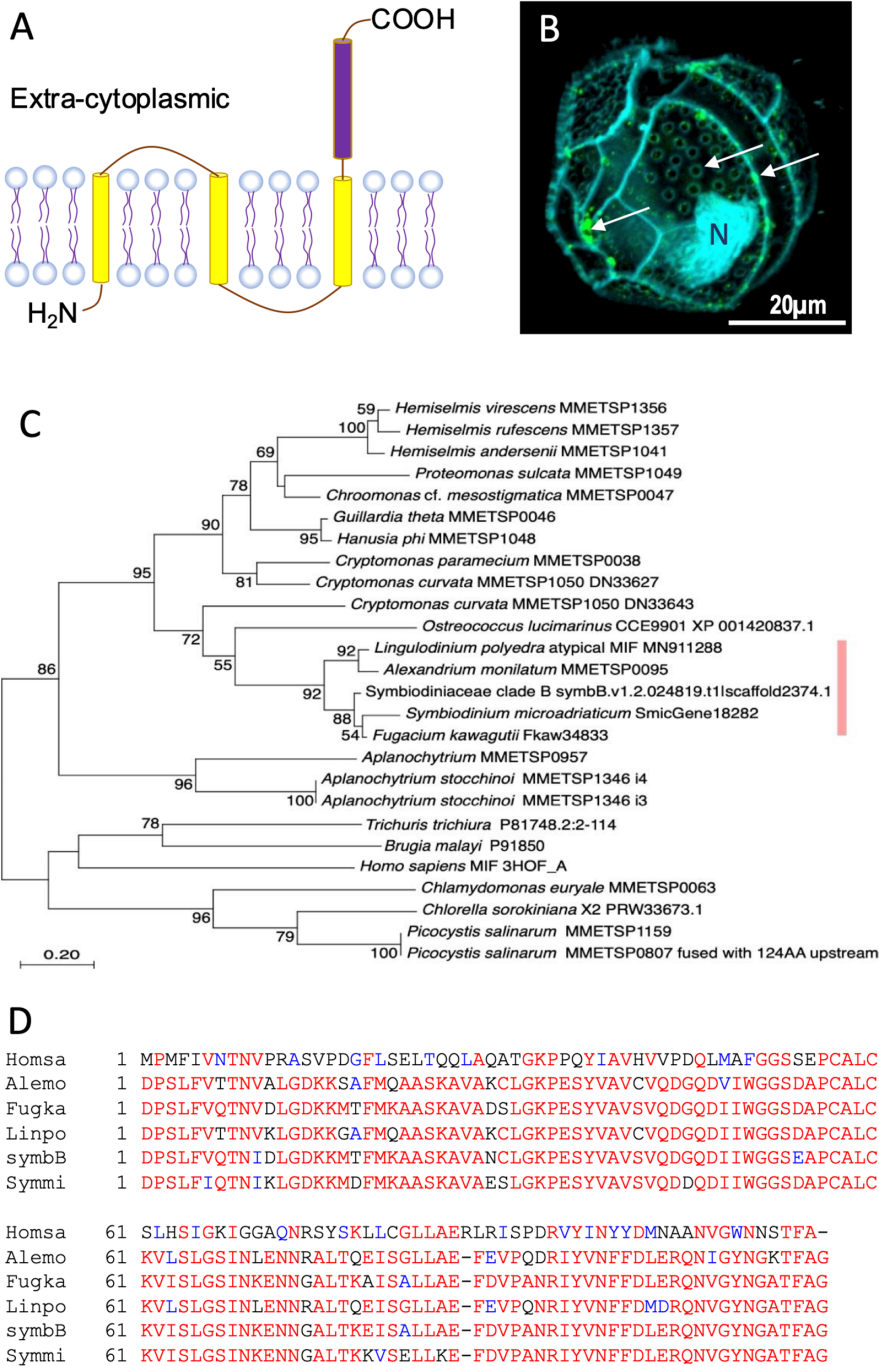
Macrophage migration inhibitory factors (MIF) detected in dinoflagellates. **A** Schematic of MIF structure. **B** Cell surface localization of MIF in *L. polyedra* (green fluorescence pointed by arrows). From Jaouannet et al. 2020 [205]. **C** Phylogenetic tree showing clustering of dinoflagellate MIF with homologs from prasinophytes and other lineages. **D** Alignment of dinoflagellate MIFs with humans MIF showing high similarity. Homsa, Homo sapiens; Alemo, *Alexandrium monilatum*; Fugka, Fugacium kawagutii; Linpo, *Lingulodinium polyedra*; SymbB, Breviolum sp.; Symmi, *Symbiodinium microadriaticum*

A MIF-like protein was detected in *L. polyedrum* (LpMIF), but instead of a soluble single-domain protein as a typical MIF is, LpMIF is a transmembrane protein with a cytosolic domain [205]. The MIF domain is localized in cell wall-associated membranes, vesicular bodies, and membranes of extracellular vesicles accumulating at the secretory pores of the cells (Fig. 11A, B). Tblastn analysis using human MIF as query against the MMETSP database (E-value e-20 cutoff) yielded 92 hits, 28 from dinoflagellates including *P. shikokuense*, *Alexandrium* spp., Symbiodiniaceae species, *Karenia brevis*, *Karlodinium veneficum*, and *Heterocapsa triquetra*. Phylogenetic analysis shows it is closely related to a prasinophyte homolog within a clade containing cryptophytes (Fig. 11C). Most dinoflagellate hits align with human MIF (hsMIF) from position 2 to 115, with some being shorter due to incompleteness (Fig. 11D).

Pfam search confirms these dinoflagellate MIF sequences contain the MIF functional domain. KEGG analysis indicates they are phenylpyruvate tautomerase (EC 5.3.2.1), the second member of the MIF superfamily, involved in Tyrosine and Phenylalanine metabolism. Under biotic stress, *L. polyedra* shows decreased MIF gene expression and reduced protein levels on the cell surface, suggesting this MIF may be involved in intercellular communication [205] or sensing of environmental stimuli.

#### RACK1 and Hemerythrin-like protein

Islas-Flores and colleagues constructed yeast-two hybrid system cDNA libraries for *Symbiodinium microadriaticum* based on DinoSL and the SMART technology [206]. Significantly more cDNAs were retrieved from the DinoSL-based libraries than the SMART-based libraries, demonstrating the advantage of DinoSL for constructing dinoflagellate full-length cDNA libraries. More importantly, using the yeast strain harboring *Symbiodinium* RACK1 (SmicRACK1) as bait and the DinoSL-based library prepared with the other yeast strain as the prey, the yeast-two hybrid system showed the interaction between SymicRACK1 and hemerythrin-like protein, suggesting the latter being the ligand of the former. RACK1 is a member of the WD-repeat protein family, sharing homology with G-protein β subunit (Gβ) and featuring a seven-bladed β-propeller structure for protein binding [207]. WD-repeat proteins, including RACK1, typically serve as scaffolds for protein complexes. They often interact with multiple partners, coordinating signaling events across various pathways and contributing to the spatiotemporal organization of cellular processes. As such, RACK1 functions in shuttling, anchoring, and stabilizing proteins within the cell, interacting with ribosomal machinery, cell surface receptors, and nuclear proteins, influencing various cellular pathways and functions [207]. Hemerythrin is an oxygen-carrier non-heme protein responsible for oxygen transport in several marine invertebrates and worms [208, 209] but also functions in innate immunity and anterior tissue regeneration in certain worms [210]. The hemerythrin-encoding gene has also been found in numerous bacterial, archaeal, and eukaryotic genomes [209]. Besides O_2_ transport, hemerythrin proteins can serve in signal transduction, phosphorelay regulation, abiotic resistance, and protein binding [208]. The specific roles of SmicRACK1 and hemerythrin-like protein in *S. microadriaticum* remain to be determined.

#### Abscisic acid 8’-hydroxylase

Abscisic acid 8’-hydroxylase (EC 1.14.13.93) belongs to the family of oxidoreductase, and it catalyzes the oxidative degradation of abscisic acid (ABA). As a member of the cytochrome P450 monooxygenase family [211], this enzyme is involved in the regulation of germination and dormancy of plant seeds [212] and participates in carotenoid biosynthesis. In dinoflagellates, the encoding gene of this enzyme was shown to be upregulated in cysts, suggesting its role in encystment and maintenance of dormancy [213]. Besides, upregulated expression of this gene was found during the initial response to heat stress in a *Cladocopium* species [214].

#### Small heat shock proteins

sHSPs are ancient, universal molecular chaperones involved in cellular stress response [215, 216]. They have a molecular weight of 12–43 kDa and a tripartite domain architecture, consisting of a conserved alpha-crystallin domain (ACD) and variable C and N terminal extensions [217].

Recent research has revealed new information on sHSP gene sequences and stress-dependent expression profiles in dinoflagellates [218]. Using DinoSL as a selective tool, Deng et al. (2021) [218] constructed dinoflagellate-specific full-length cDNA libraries from marine sediment-derived RNA samples. They found sHSPs with A-X-X-X-D/N/S/H/R-G-V-L in the typical conserved A-X-X-X-N-G-V-L motif. Active expression of these sHSPs in sediment suggests their potential role in maintaining dinoflagellate cyst dormancy.

#### Meiosis genes

Sexual reproduction is widespread among eukaryotes and serves as a mechanism for chromosome recombination, thereby generating genetic diversity [219–221]. Dinoflagellates typically have a haplontic life cycle, consisting of haploid asexual and diploid sexual stages. Meiosis is one of the hallmarks of sexuality, and its regulating genes only began to be identified in dinoflagellates. From genomes and transcriptomes, a range of genes (31–51) with homology to meiosis genes in model organisms have been identified [18, 222, 223]. These include most of the 12 core meiosis genes previously developed for eukaryotes [224] and homologs of yeast meiosis regulating genes such as MEI2 and several meiotically upregulated genes (Table S8 in Lin et al. 2015 [18]). However, securing, which normally serve in preventing the transition from metaphase to anaphase by inhibiting the protease separin, and some components of the cohesion complex with the function to hold homologous chromosomes together are absent [222]. The absence of cohesion complex elements is consistent with more recent findings suggesting that dinoflagellates may have lost the canonical synaptonemal complex [225].

Syngamy is another important hallmark of sexuality. In a transcriptomic study of *P. cordatum*, HAP2 was identified, along with MEI2, MutS2, and several other meiosis-associated genes [226]. HAP2 (hapless 2) participates in the fusion of cell membranes [227].

In dinoflagellates, sexual reproduction is known to occur under adverse environmental conditions and lead to encystment for survival (‘sex for encystment’) (review in [228]). However, increasing laboratory observations indicate that sexual reproduction can also produce vegetative cells, bypassing encystment [229]. Recent natural bloom transcriptomic studies suggest that sexual reproduction in dinoflagellates may aid in bloom proliferation or extension (‘sex for proliferation’). Specifically, genes specific to or associated with meiosis were significantly upregulated during blooms, unexpected for the ‘sex for encystment’ scenario [100, 166]. Integrating transcriptomic data from natural blooms and laboratory cultures of *P. shikokuense*, *Scrippsiella acuminata*, and *Karenia mikimotoi*, 28 meiosis genes were identified and found to be highly expressed during blooms or cyst germination. Key genes include MEI2, initially identified in yeast for initiating meiosis. RAD21, SMC3, and MEI2 are linked to bloom-promoting sex, HOP2 and MSH4 to cyst germination, while SPO11, MND1, and DMC1 are common to both cyst-forming and non-encysting sex [100]. Verification of these findings could provide markers for distinguishing different sexual reproduction ecotypes in dinoflagellates.

#### Response to temperature variation

Temperature responses have not received as much research attention as nutrient responses for molecular understanding. Recently, Zhang et al. (2022) [230] investigated proteomic and metabolomic changes in the HAB species *P. shikokuense* across four temperatures: optimal (25 °C), supra-optimal (28 °C), and sub-optimal (19 °C and 22 °C). They observed decreases in particulate organic carbon and nitrogen (POC, PON) as temperature increased, with an increased POC/PON ratio at higher temperatures, except at 28 °C. These results suggest a higher cell division rate and photosynthetic carbon fixation at higher temperatures. However, at the supra-optimal temperature, cells increased synthesis of light harvesting, photoreaction, and protein homeostasis related proteins. Sub-optimal temperatures upregulated glutathione (an antioxidant), transcription, and lipid biosynthesis, compensating for decreased translation efficiency and cell membrane fluidity. At 19 °C, there was an increase in nitrate reduction and nitrogen flux towards asparagine, aspartic acid, and glutamine, with the accumulation of glutathione, glutarate semialdehyde, and 5-KETE.

Interestingly, the metabolomic data of the study showed a significant increase in a sulfinpyrazone-like metabolite at 19 °C compared to 25 °C (13-fold) and 28 °C (26-fold). Sulfinpyrazone (C23H20N2O3S) is a medicine to treat gout patients as it inhibits reabsorption (transport) of uric acid and enhances its urine excretion [231]. Whether this compound blocks intracellular cross-membrane trafficking of organic nitrogen molecules or is an accumulated intermediate at lower temperatures remains to be investigated.

Another study on *P. cordatum* revealed differential gene cluster responses between mRNA editing and alternative splicing (exon usage) to temperature changes between 20 °C and 26 °C [22]. Further verification could indicate a novel mechanism of gene regulation. Additionally, *P. glacialis* is an ideal model for understanding cold and darkness adaptation, having acquired genes from bacteria for ice-binding proteins. This species shows enhanced transcriptional responses through unidirectional, tandem duplication of single-exon genes involved in cold survival and low-light adaptation [21].

### Future prospect

Dinomics has lagged behind the omics research of other major algal groups, but a decade of dedicated effort has produced remarkable insights. Many questions in dinoflagellate biology remain to be explored through broader and deeper omics research complemented by other cutting-edge technologies. Some of the key research gaps are discussed below.

*A finished dinoflagellate genome* is urgently needed to fully understand genome architecture and coding landscapes. The genomes sequenced so far do not exhibit the previously suspected high complexity, but this may change with the sequencing of larger genomes. Furthermore, accurate gene prediction for dinoflagellates likely requires substantial improvement of algorithms. Additionally, no clear evidence of genome duplication has been found, potentially due to the bias towards smaller genomes. The dual-peak k-mer profile in *P. glacialis* and chromosomal pairing in *P. lunula* suggest that genome duplication might be prevalent in large dinoflagellate genomes. Sequencing and achieving high-quality assemblies for ecologically important species like *Alexandrium catenella* (Group I) would be fruitful.

*Tomographical Imaging of Chromosomal Architecture* harnessed by combining cutting-edge imaging technology with omics, such as Cryo-EM analyses [158, 173], can address many architectural questions of dinoflagellate chromosomes. It remains unclear how the extreme ‘chimeric’ repertoire of nuclear proteins shapes the constantly condensed chromosomes, regulates DNA duplication, and coordinates gene expression in response to environmental changes. Recent transcriptomic and genome methylation data suggest that dinoflagellates may use nucleosomal arrays to regulate gene transcription [89, 90]. In situ nuclear imaging of samples from different physiological states could shed light on these mysteries.

*Functional Genetic Research* to accelerate functional characterization of the > 50% unannotated dinoflagellate genes is crucial to advance dinoflagellate biology. Additionally, a substantial fraction of “annotated” genes have only weak matches to known genes, and their exact functions need to be experimentally verified. Accessible functional genetic tools need to be established. Gene transformation began in the 1990s and continued through the 2000s, but reproducible success has been limited [232–234]. Gene knockdown has been explored for a few genes, e.g., Rubisco, rhodopsin [101], cellulose synthase CesA1 [235], and eukaryotic translation initiation factor eIF4e [236]. Gene knockout studies using CRISPR/Cas technology have yet to emerge.

#### Multi-Omic Approach is necessary to Addressing Gene Regulation

Only by systematic investigation can transcriptional, translational, and post-translational gene regulation be unequivocally determined. The long-held notion of limited transcriptional regulation in dinoflagellates may change, as increasing environment-responsive genes have been reported. Translational and posttranslational regulation, including epigenetic mechanisms, need to be investigated simultaneously. Transcriptomics, proteomics, metabolomics, and epigenomics should be pursued together. Collaboration among experts in these subdisciplines is crucial to tackle the same species in a coordinated manner.

*Single-Cell Transcriptomics *is widely used for marine prokaryotes, but needs to be explored more extensively for eukaryotes [237]. Applying this technique to sequence the transcriptome of cells from the field has recently led to the discovery of new dinoflagellate species [238]. Single-cell sequencing of sorted cells from different spatial positions in a natural bloom can potentially pinpoint the major drivers of the bloom and the range of physiologies occurring within the population.

#### Integrative Molecular Ecological Framework should be developed

Translating genomic understanding of dinoflagellates into insights about their ecological behaviors in the natural environment requires an integrative framework. Many fundamental ecological questions cannot be answered with omics data or ecological data alone. For example, understanding how dinoflagellates proliferate to form blooms or what triggers coral bleaching requires a quantitative model relating gene expression to physiological rates or biotic interactions. A standardized approach will enable cross-system comparisons. Data and insights from laboratory cultures are foundational to understanding the dynamics and dominance of dinoflagellate species in natural plankton communities. However, in situ studies should be conducted, and such work should be set on an integrative framework from multiple perspectives such as energy (E) and nutrient (N) acquisition, defense (D) against biotic and abiotic stresses, and sexual and asexual reproduction (S) [101, 178]. This ENDS framework has initially facilitated comparisons of how different phytoplankton lineages perform with various nutritional strategies and understand the factors regulating harmful algal bloom outbreaks [82, 102, 178, 239].

## Data Availability

No datasets were generated or analysed during the current study.

## References

[1] Hackett JD, Anderson DM, Erdner DL, Bhattacharya D. Dinoflagellates: a remarkable evolutionary experiment. Am J Bot. 2004;91(10):1523–34. 21652307 10.3732/ajb.91.10.1523

[2] Lin S. Genomic understanding of dinoflagellates. Res Microbiol. 2011;162(6):551–69. 21514379 10.1016/j.resmic.2011.04.006

[3] Fensome RA. A classification of living and fossil dinoflagellates. Micropaleontology Spec Publ. 1993;7:351.

[4] Gómez F. A checklist and classification of living dinoflagellates (Dinoflagellata, Alveolata). Cicimar Oceánides. 2012;27(1):65–140.

[5] Logares R, Shalchian-Tabrizi K, Boltovskoy A, Rengefors K. Extensive dinoflagellate phylogenies indicate infrequent marine–freshwater transitions. Mol Phylogenet Evol. 2007;45(3):887–903. 17928239 10.1016/j.ympev.2007.08.005

[6] Annenkova NV, Hansen G, Rengefors K. Closely related dinoflagellate species in vastly different habitats–an example of a marine–freshwater transition. Eur J Phycol. 2020;55(4):478–89.

[7] Spector DL. Dinoflagellate nuclei. Dinoflagellates. 1984;1:107–47.

[8] Wisecaver JH, Hackett JD. Dinoflagellate genome evolution. Annu Rev Microbiol. 2011;65:369–87 65, 2011. 21682644 10.1146/annurev-micro-090110-102841

[9] Bachvaroff TR. A precedented nuclear genetic code with all three termination codons reassigned as sense codons in the syndinean Amoebophrya sp. ex Karlodinium Veneficum. PLoS ONE. 2019;14(2): e0212912. 30818350 10.1371/journal.pone.0212912 PMC6394959

[10] John U, Lu Y, Wohlrab S, Groth M, Janouškovec J, Kohli GS, et al. An aerobic eukaryotic parasite with functional mitochondria that likely lacks a mitochondrial genome. Sci Adv. 2019;5(4): eaav1110. 31032404 10.1126/sciadv.aav1110 PMC6482013

[11] Farhat S, Le P, Kayal E, Noel B, Bigeard E, Corre E, et al. Rapid protein evolution, organellar reductions, and invasive intronic elements in the marine aerobic parasite dinoflagellate Amoebophrya spp. BMC Biol. 2021;19:1–21. 33407428 10.1186/s12915-020-00927-9 PMC7789003

[12] Beedessee G, Kubota T, Arimoto A, Nishitsuji K, Waller RF, Hisata K, et al. Integrated omics unveil the secondary metabolic landscape of a basal dinoflagellate. BMC Biol. 2020;18:1–16. 33050904 10.1186/s12915-020-00873-6 PMC7557087

[13] Shoguchi E, Shinzato C, Kawashima T, Gyoja F, Mungpakdee S, Koyanagi R, et al. Draft assembly of the Symbiodinium Minutum nuclear genome reveals dinoflagellate gene structure. Curr Biol. 2013;23(15):1399–408. 23850284 10.1016/j.cub.2013.05.062

[14] Liu H, Stephens TG, González-Pech RA, Beltran VH, Lapeyre B, Bongaerts P, et al. Symbiodinium genomes reveal adaptive evolution of functions related to coral-dinoflagellate symbiosis. Commun Biolog. 2018;1(1):95. 10.1038/s42003-018-0098-3 PMC6123633 30271976

[15] Chen Y, Shah S, Dougan KE, van Oppen MJ, Bhattacharya D, Chan CX. Improved cladocopium goreaui genome assembly reveals features of a facultative coral symbiont and the complex evolutionary history of dinoflagellate genes. Microorganisms. 2022;10(8):1662. 36014080 10.3390/microorganisms10081662 PMC9412976

[16] Shoguchi E, Beedessee G, Hisata K, Tada I, Narisoko H, Satoh N, et al. A new dinoflagellate genome illuminates a conserved gene cluster involved in sunscreen biosynthesis. Genome Biol Evol. 2021;13(2):evaa235. 33146374 10.1093/gbe/evaa235 PMC7875005

[17] Shah S, Dougan KE, Chen Y, Lo R, Laird G, Fortuin MD, et al. Massive genome reduction predates the divergence of Symbiodiniaceae dinoflagellates. ISME J. 2024;18(1):wrae059. 38655774 10.1093/ismejo/wrae059 PMC11114475

[18] Lin S, Cheng S, Song B, Zhong X, Lin X, Li W, et al. The Symbiodinium kawagutii genome illuminates dinoflagellate gene expression and coral symbiosis. Science. 2015;350(6261):691–4. 26542574 10.1126/science.aad0408

[19] Li T, Yu L, Song B, Song Y, Li L, Lin X, et al. Genome improvement and core gene set refinement of Fugacium Kawagutii. Microorganisms. 2020;8(1): 102. 31940756 10.3390/microorganisms8010102 PMC7023079

[20] Gornik SG, Febrimarsa Cassin AM, MacRae JI, Ramaprasad A, Rchiad Z, et al. Endosymbiosis undone by stepwise elimination of the plastid in a parasitic dinoflagellate. Proc Natl Acad Sci. 2015;112(18):5767–72. 25902514 10.1073/pnas.1423400112 PMC4426444

[21] Stephens TG, González-Pech RA, Cheng Y, Mohamed AR, Burt DW, Bhattacharya D, et al. Genomes of the dinoflagellate polarella glacialis encode tandemly repeated single-exon genes with adaptive functions. BMC Biol. 2020;18:1–21. 32448240 10.1186/s12915-020-00782-8 PMC7245778

[22] Dougan KE, Deng Z-L, Wöhlbrand L, Reuse C, Bunk B, Chen Y, et al. Multi-omics analysis reveals the molecular response to heat stress in a red tide dinoflagellate. Genome Biol. 2023;24(1):265. 37996937 10.1186/s13059-023-03107-4 PMC10666404

[23] González-Pech RA, Stephens TG, Chen Y, Mohamed AR, Cheng Y, Shah S, et al. Comparison of 15 dinoflagellate genomes reveals extensive sequence and structural divergence in family Symbiodiniaceae and Genus Symbiodinium. BMC Biol. 2021;19:1–22. 33849527 10.1186/s12915-021-00994-6 PMC8045281

[24] Aranda M, Li Y, Liew YJ, Baumgarten S, Simakov O, Wilson MC, et al. Genomes of coral dinoflagellate symbionts highlight evolutionary adaptations conducive to a symbiotic lifestyle. Sci Rep. 2016;6(1): 39734. 28004835 10.1038/srep39734 PMC5177918

[25] Shoguchi E, Beedessee G, Tada I, Hisata K, Kawashima T, Takeuchi T, et al. Two divergent Symbiodinium genomes reveal conservation of a gene cluster for sunscreen biosynthesis and recently lost genes. BMC Genomics. 2018;19:1–11. 29898658 10.1186/s12864-018-4857-9 PMC6001144

[26] Zheng S, Wang G, Lin S. Heat shock effects and population survival in the polar dinoflagellate Polarella Glacialis. J Exp Mar Biol Ecol. 2012;438:100–8.

[27] Lin S, Zhang H, Hou Y, Zhuang Y, Miranda L. High-level diversity of dinoflagellates in the natural environment, revealed by assessment of mitochondrial cox1 and cob genes for dinoflagellate DNA barcoding. Appl Environ Microbiol. 2009;75(5):1279–90. 19114529 10.1128/AEM.01578-08 PMC2648164

[28] Nand A, Zhan Y, Salazar OR, Aranda M, Voolstra CR, Dekker J. Genetic and spatial organization of the unusual chromosomes of the dinoflagellate Symbiodinium microadriaticum. Nat Genet. 2021;53(5):618–29. 33927399 10.1038/s41588-021-00841-y PMC8110479

[29] Marinov GK, Trevino AE, Xiang T, Kundaje A, Grossman AR, Greenleaf WJ. Transcription-dependent domain-scale three-dimensional genome organization in the dinoflagellate Breviolum Minutum. Nat Genet. 2021;53(5):613–7. 33927397 10.1038/s41588-021-00848-5 PMC8110477

[30] Sigee DC. The dinoflagellate chromosome. Adv Bot Res. 1986;12:205–64.

[31] Zaheri B, Dagenais-Bellefeuille S, Song B, Morse D. Assessing transcriptional responses to light by the dinoflagellate Symbiodinium. Microorganisms. 2019;7(8): 261. 31416260 10.3390/microorganisms7080261 PMC6723345

[32] Blank RJ, Trench RK. Speciation and symbiotic dinoflagellates. Science. 1985;229(4714):656–8. 17739379 10.1126/science.229.4714.656

[33] Lin S, Song B, Morse D. Spatial organization of dinoflagellate genomes: novel insights and remaining critical questions. J Phycol. 2021;57(6):1674–8. 34389979 10.1111/jpy.13206

[34] Alverca E, Cuadrado A, Jouve N, Franca S, Moreno Díaz, de la Espina S. Telomeric DNA localization on dinoflagellate chromosomes: structural and evolutionary implications. Cytogenet Genome Res. 2007;116(3):224–31. 17317964 10.1159/000098191

[35] Fojtová M, Wong JT, Dvořáčková M, Yan KT, Sýkorová E, Fajkus J. Telomere maintenance in liquid crystalline chromosomes of dinoflagellates. Chromosoma. 2010;119:485–93. 20369246 10.1007/s00412-010-0272-y

[36] Cuadrado Á, Figueroa RI, Sixto M, Bravo I, De Bustos A. First record of the spatial organization of the nucleosome-less chromatin of dinoflagellates: the nonrandom distribution of microsatellites and bipolar arrangement of telomeres in the nucleus of Gambierdiscus australes (Dinophyceae). J Phycol. 2022;58(2):297–307. 35038777 10.1111/jpy.13236

[37] Cuadrado Á, De Bustos A, Figueroa RI. Chromosomal markers in the genus Karenia: towards an understanding of the evolution of the chromosomes, life cycle patterns and phylogenetic relationships in dinoflagellates. Sci Rep. 2019;9(1):3072. 30816125 10.1038/s41598-018-35785-7 PMC6395649

[38] Pombo A, Dillon N. Three-dimensional genome architecture: players and mechanisms. Nat Rev Mol Cell Biol. 2015;16(4):245–57. 25757416 10.1038/nrm3965

[39] Roy S, Morse D. A full suite of histone and histone modifying genes are transcribed in the dinoflagellate Lingulodinium. PLoS ONE. 2012;7(4):e34340. 22496791 10.1371/journal.pone.0034340 PMC3319573

[40] Soyer M-O, Haapala O. Electron microscopy of RNA in dinoflagellate chromosomes. Histochemistry. 1974;42(3):239–46. 4477158 10.1007/BF00492656

[41] Veldhuis MJ, Cucci TL, Sieracki ME. Cellular DNA content of marine phytoplankton using two new fluorochromes: taxonomic and ecological implications 1. J Phycol. 1997;33(3):527–41.

[42] LaJeunesse TC, Lambert G, Andersen RA, Coffroth MA, Galbraith DW. Symbiodinium (pyrrhophyta) genome sizes (DNA content) are smallest among dinoflagellates 1. J Phycol. 2005;41(4):880–6.

[43] Janouškovec J, Gavelis GS, Burki F, Dinh D, Bachvaroff TR, Gornik SG, et al. Major transitions in dinoflagellate evolution unveiled by phylotranscriptomics. Proc Natl Acad Sci. 2017;114(2):E171–80. 28028238 10.1073/pnas.1614842114 PMC5240707

[44] Wolfe KH, Shields DC. Molecular evidence for an ancient duplication of the entire yeast genome. Nature. 1997;387(6634):708–13. 9192896 10.1038/42711

[45] Crow KD, Wagner GP. What is the role of genome duplication in the evolution of complexity and diversity? Mol Biol Evol. 2005;23(5):887–92. 16368775 10.1093/molbev/msj083

[46] Seo KS, Fritz L. Karyology of a marine non-motile dinoflagellate, Pyrocystis lunula. Hydrobiologia. 2006;563:289–96.

[47] Chen J, Lu L, Robb SM, Collin M, Okumoto Y, Stajich JE, et al. Genomic diversity generated by a transposable element burst in a rice recombinant inbred population. Proc Natl Acad Sci. 2020;117(42):26288–97. 33020276 10.1073/pnas.2015736117 PMC7584900

[48] Hou Y, Ji N, Zhang H, Shi X, Han H, Lin S. Genome size-dependent PCNA gene copy number in dinoflagellates and molecular evidence of retroposition as a major evolutionary mechanism. J Phycol 2018;55(1):37-46. 30468510 10.1111/jpy.12815

[49] Song B, Morse D, Song Y, Fu Y, Lin X, Wang W, et al. Comparative genomics reveals two major bouts of gene retroposition coinciding with crucial periods of Symbiodinium evolution. Genome Biol Evol. 2017;9(8):2037–47. 28903461 10.1093/gbe/evx144 PMC5585692

[50] Galindo-González L, Mhiri C, Deyholos MK, Grandbastien M-A. LTR-retrotransposons in plants: engines of evolution. Gene. 2017;626:14–25. 28476688 10.1016/j.gene.2017.04.051

[51] Chen JE, Cui G, Wang X, Liew YJ, Aranda M. Recent expansion of heat-activated retrotransposons in the coral symbiont Symbiodinium microadriaticum. ISME J. 2018;12(2):639–43. 29053149 10.1038/ismej.2017.179 PMC5776459

[52] de Mendoza A, Bonnet A, Vargas-Landin DB, Ji N, Li H, Yang F, et al. Recurrent acquisition of cytosine methyltransferases into eukaryotic retrotransposons. Nat Commun. 2018;9(1):1341. 29632298 10.1038/s41467-018-03724-9 PMC5890265

[53] Hu L, Li N, Zhang Z, Meng X, Dong Q, Xu C, et al. CG hypomethylation leads to complex changes in DNA methylation and transpositional burst of diverse transposable elements in callus cultures of rice. Plant J. 2020;101(1):188–203. 31529551 10.1111/tpj.14531

[54] Collens AB, Katz LA. Opinion: genetic conflict with mobile elements drives eukaryotic genome evolution, and perhaps also eukaryogenesis. J Hered. 2021;112(1):140–4. 33538295 10.1093/jhered/esaa060 PMC7953837

[55] Zhou W, Liang G, Molloy PL, Jones PA. DNA methylation enables transposable element-driven genome expansion. Proc Natl Acad Sci. 2020;117(32):19359–66. 32719115 10.1073/pnas.1921719117 PMC7431005

[56] Bhaud Y, Salmon J-M, Soyer-Gobillard M-O. The complex cell cycle of the dinoflagellate protoctist Crypthecodinium cohnii as studied in vivo and by cytofluorimetry. J Cell Sci. 1991;100(3):675–82.

[57] Allen J, Roberts TM, Loeblich AR III, Klotz LC. Characterization of the DNA from the dinoflagellate Crypthecodinium cohnii and implications for nuclear organization. Cell. 1975;6(2):161–9. 1237354 10.1016/0092-8674(75)90006-9

[58] Jaeckisch N, Yang I, Wohlrab S, Glöckner G, Kroymann J, Vogel H, et al. Comparative genomic and transcriptomic characterization of the toxigenic marine dinoflagellate Alexandrium ostenfeldii. PLoS ONE. 2011;6(12):e28012. 22164224 10.1371/journal.pone.0028012 PMC3229502

[59] Wisecaver JH, Brosnahan ML, Hackett JD. Horizontal gene transfer is a significant driver of gene innovation in dinoflagellates. Genome Biol Evol. 2013;5(12):2368–81. 24259313 10.1093/gbe/evt179 PMC3879968

[60] Van Etten J, Bhattacharya D. Horizontal gene transfer in eukaryotes: not if, but how much? Trends Genet. 2020;36(12):915–25. 33012528 10.1016/j.tig.2020.08.006

[61] Nosenko T, Bhattacharya D. Horizontal gene transfer in chromalveolates. BMC Evol Biol. 2007;7:1–18. 17894863 10.1186/1471-2148-7-173 PMC2064935

[62] Mackiewicz P, Bodył A, Moszczyński K. The case of horizontal gene transfer from bacteria to the peculiar dinoflagellate plastid genome. Mob Genetic Elem. 2013;3(4):e25845. 10.4161/mge.25845 PMC3812789 24195014

[63] Morse D, Salois P, Markovic P, Hastings JW. A nuclear-encoded form II RuBisCO in dinoflagellates. Science. 1995;268(5217):1622–4. 7777861 10.1126/science.7777861

[64] Whitney SM, Shaw DC, Yellowlees D. Evidence that some dinoflagellates contain a ribulose-1,5-bisphosphate carboxylase / oxygenase related to that of the α-proteobacteria. Proc Biol Sci. 1995;259(1356):271–5. 7740046 10.1098/rspb.1995.0040

[65] Rowan R, Whitney SM, Fowler A, Yellowlees D. Rubisco in Marine symbiotic dinoflagellates: form II enzymes in eukaryotic oxygenic phototrophs encoded by a nuclear multigene family. Plant Cell. 1996;8(3):539–53. 8721755 10.1105/tpc.8.3.539 PMC161119

[66] Béjà O, Aravind L, Koonin EV, Suzuki MT, Hadd A, Nguyen LP, et al. Bacterial rhodopsin: evidence for a New type of Phototrophy in the Sea. Science. 2000;289(5486):1902–6. 10988064 10.1126/science.289.5486.1902

[67] Rozenberg A, Inoue K, Kandori H, Béjà O. Microbial rhodopsins: the last two decades. Annu Rev Microbiol. 2021;75(1):427–47. 34343014 10.1146/annurev-micro-031721-020452

[68] Lin S, Zhang H, Zhuang Y, Tran B, Gill J. Spliced leader–based metatranscriptomic analyses lead to recognition of hidden genomic features in dinoflagellates. Proc Natl Acad Sci. 2010;107(46):20033–8. 21041634 10.1073/pnas.1007246107 PMC2993343

[69] Marchetti A, Schruth DM, Durkin CA, Parker MS, Kodner RB, Berthiaume CT, et al. Comparative metatranscriptomics identifies molecular bases for the physiological responses of phytoplankton to varying iron availability. Proc Natl Acad Sci. 2012;109(6):E317–25. 22308424 10.1073/pnas.1118408109 PMC3277525

[70] Guo Z, Zhang H, Lin S. Light-promoted rhodopsin expression and starvation survival in the marine dinoflagellate Oxyrrhis marina. PLoS ONE. 2014;9(12): e114941. 25506945 10.1371/journal.pone.0114941 PMC4266641

[71] Shi X, Li L, Guo C, Lin X, Li M, Lin S. Rhodopsin gene expression regulated by the light dark cycle, light spectrum and light intensity in the dinoflagellate Prorocentrum. Front Microbiol. 2015;6:555. 26082770 10.3389/fmicb.2015.00555 PMC4451421

[72] Shi X, Lin X, Li L, Li M, Palenik B, Lin S. Transcriptomic and microRNAomic profiling reveals multi-faceted mechanisms to cope with phosphate stress in a dinoflagellate. ISME J. 2017;11(10):2209–18. 28548660 10.1038/ismej.2017.81 PMC5607363

[73] Ma M, Shi X, Lin S. Heterologous expression and cell membrane localization of dinoflagellate opsins (rhodopsin proteins) in mammalian cells. Mar Life Sci Technol. 2020;2:302–8.

[74] Slamovits CH, Okamoto N, Burri L, James ER, Keeling PJ. A bacterial proteorhodopsin proton pump in marine eukaryotes. Nat Commun. 2011;2(1):183. 21304512 10.1038/ncomms1188

[75] Rhiel E, Hoischen C, Westermann M. Rhodopsins build up the birefringent bodies of the dinoflagellate Oxyrrhis marina. Protoplasma. 2022;259(4):1047-60. 10.1007/s00709-021-01717-y PMC9184458 34738175

[76] Westermann M, Hoischen C, Wöhlbrand L, Rabus R, Rhiel E. Light and prey influence the abundances of two rhodopsins in the dinoflagellate Oxyrrhis marina. Protoplasma. 2023;260(2):529–44. 35871098 10.1007/s00709-022-01795-6 PMC9931815

[77] Rozenberg A, Kaczmarczyk I, Matzov D, Vierock J, Nagata T, Sugiura M, et al. Rhodopsin-bestrophin fusion proteins from unicellular algae form gigantic pentameric ion channels. Nat Struct Mol Biol. 2022;29(6):592–603. 35710843 10.1038/s41594-022-00783-x

[78] Nagasaki K. Dinoflagellates, diatoms, and their viruses. J Microbiol. 2008;46(3):235–43. 18604491 10.1007/s12275-008-0098-y

[79] Correa AM, Welsh RM, Vega Thurber RL. Unique nucleocytoplasmic dsDNA and + ssRNA viruses are associated with the dinoflagellate endosymbionts of corals. ISME J. 2013;7(1):13–27. 22791238 10.1038/ismej.2012.75 PMC3526182

[80] Veglia AJ, Bistolas KS, Voolstra CR, Hume BC, Ruscheweyh H-J, Planes S, et al. Endogenous viral elements reveal associations between a non-retroviral RNA virus and symbiotic dinoflagellate genomes. Commun Biology. 2023;6(1):566. 10.1038/s42003-023-04917-9 PMC10235124 37264063

[81] Wang J, Li L, Lin S. Active viral infection during blooms of a dinoflagellate indicates dinoflagellate-viral co-adaptation. Appl Environ Microbiol. 2023;89(11):e01156-01123. 37874280 10.1128/aem.01156-23 PMC10686096

[82] Li X, Li Z, Wang F, Zhao S, Xu C, Mao Z, et al. Structures and organizations of PSI–AcpPCI supercomplexes from red tidal and coral symbiotic photosynthetic dinoflagellates. Proc Natl Acad Sci. 2024;121(7): e2315476121. 38319970 10.1073/pnas.2315476121 PMC10873603

[83] Gornik SG, Ford KL, Mulhern TD, Bacic A, McFadden GI, Waller RF. Loss of nucleosomal DNA condensation coincides with appearance of a novel nuclear protein in dinoflagellates. Curr Biol. 2012;22(24):2303–12. 23159597 10.1016/j.cub.2012.10.036

[84] Gornik SG, Hu I, Lassadi I, Waller RF. The biochemistry and evolution of the dinoflagellate nucleus. Microorganisms. 2019;7(8):245. 31398798 10.3390/microorganisms7080245 PMC6723414

[85] Kearns L, Sigee D. The occurrence of period IV elementsin dinoflagellate chromatin: an X-ray microanalytical study. J Cell Sci. 1980;46(1):113–27. 7194876 10.1242/jcs.46.1.113

[86] Herzog M, Soyer M. The native structure of dinoflagellate chromosomes and their stabilization by Ca2 + and Mg2 + cations. Eur J Cell Biol. 1983;30(1):33–41. 6682763

[87] Levi-Setti R, Gavrilov KL, Rizzo PJ. Divalent cation distribution in dinoflagellate chromosomes imaged by high-resolution ion probe mass spectrometry. Eur J Cell Biol. 2008;87(12):963–76. 18707794 10.1016/j.ejcb.2008.06.002

[88] Beauchemin M, Morse D. A proteomic portrait of dinoflagellate chromatin reveals abundant RNA-binding proteins. Chromosoma. 2018;127:29–43. 28852823 10.1007/s00412-017-0643-8

[89] Marinov GK, Lynch M. Diversity and divergence of dinoflagellate histone proteins. G3. 2016;6(2):397–422. 10.1534/g3.115.023275 PMC4751559 26646152

[90] Marinov GK, Chen X, Swaffer MP, Xiang T, Grossman AR, Greenleaf WJ. Genome-wide distribution of 5-hydroxymethyluracil and chromatin accessibility in the Breviolum minutum genome. Genome Biol. 2024;25(1):115. 38711126 10.1186/s13059-024-03261-3 PMC11071213

[91] Chan Y-H, Wong JT. Concentration-dependent organization of DNA by the dinoflagellate histone-like protein HCc3. Nucleic Acids Res. 2007;35(8):2573–83. 17412706 10.1093/nar/gkm165 PMC1885672

[92] Wong JTY. Architectural organization of dinoflagellate liquid crystalline chromosomes. Microorganisms. 2019;7(2):27. 30678153 10.3390/microorganisms7020027 PMC6406473

[93] Irwin NA, Martin BJ, Young BP, Browne MJ, Flaus A, Loewen CJ, et al. Viral proteins as a potential driver of histone depletion in dinoflagellates. Nat Commun. 2018;9(1):1535. 29670105 10.1038/s41467-018-03993-4 PMC5906630

[94] Wang J, Li H, Li L, Wang Y, Lin S. Comparative genomics illuminates adaptive evolution of DVNP with lifestyle and with loss of histone H1 in dinoflagellates. bioRxiv. 2024;202402(09):579734.

[95] Wong JT, New D, Wong J, Hung V. Histone-like proteins of the dinoflagellate Crypthecodinium cohnii have homologies to bacterial DNA-binding proteins. Eukaryot Cell. 2003;2(3):646–50. 12796310 10.1128/EC.2.3.646-650.2003 PMC161454

[96] Mendez GS, Delwiche CF, Apt KE, Lippmeier JC. Dinoflagellate gene structure and intron splice sites in a genomic tandem array. J Eukaryot Microbiol. 2015;62(5):679–87. 25963315 10.1111/jeu.12230 PMC5032977

[97] Roy SW, Gozashti L, Bowser BA, Weinstein BN, Larue GE, Corbett-Detig R. Intron-rich dinoflagellate genomes driven by Introner transposable elements of unprecedented diversity. Curr Biol. 2023;33(1):189-96. e4. 36543167 10.1016/j.cub.2022.11.046

[98] Hou Y, Lin S. Distinct gene number-genome size relationships for eukaryotes and non-eukaryotes: gene content estimation for dinoflagellate genomes. PLoS ONE. 2009;4(9): e6978. 19750009 10.1371/journal.pone.0006978 PMC2737104

[99] Li T, Yu Y, Chen X, Lin X, Li L, Guo C, Lin S. Remarkable metabolic reconfiguration due to N deficiency and an ammonium-to-nitrate shift in the free-living Effrenium voratum (Symbiodiniaceae). J Geophys Res-Biogeochem. 2011;126:e2020JG006172. 10.1029/2020JG006172.

[100] Lin S, Yu L, Wu X, Li M, Zhang Y, Luo H, et al. Active meiosis during dinoflagellate blooms: a ‘sex for proliferation’hypothesis. Harmful Algae. 2022;118: 102307. 36195414 10.1016/j.hal.2022.102307

[101] Zhang Y, Lin X, Shi X, Lin L, Luo H, Li L, et al. Metatranscriptomic signatures associated with phytoplankton regime shift from diatom dominance to a dinoflagellate bloom. Front Microbiol. 2019;10: 590. 30967855 10.3389/fmicb.2019.00590 PMC6439486

[102] Yu L, Zhang Y, Li M, Wang C, Lin X, Li L, et al. Comparative metatranscriptomic profiling and microRNA sequencing to reveal active metabolic pathways associated with a dinoflagellate bloom. Sci Total Environ. 2020;699: 134323. 31522044 10.1016/j.scitotenv.2019.134323

[103] Li H, Li L, Yu L, Yang X, Shi X, Wang J, et al. Transcriptome profiling reveals versatile dissolved organic nitrogen utilization, mixotrophy, and N conservation in the dinoflagellate Prorocentrum shikokuense under N deficiency. Sci Total Environ. 2021;763: 143013. 33203560 10.1016/j.scitotenv.2020.143013

[104] Zhang H, Lin S. Complex gene structure of the form II rubisco in the dinoflagellate Prorocentrum minimum (dinophyceae) 1. J Phycol. 2003;39(6):1160–71.

[105] Zhang H, Hou Y, Miranda L, Campbell DA, Sturm NR, Gaasterland T, et al. Spliced leader RNA trans-splicing in dinoflagellates. Proc Natl Acad Sci. 2007;104(11):4618–23. 17360573 10.1073/pnas.0700258104 PMC1838650

[106] Hiller RG, Wrench PM, Sharples FP. The light-harvesting chlorophyll a-c-binding protein of dinoflagellates: a putative polyprotein. FEBS Lett. 1995;363(1–2):175–8. 7729542 10.1016/0014-5793(95)00297-m

[107] Li L, Hong R, Hastings JW. Three functional luciferase domains in a single polypeptide chain. Proc Natl Acad Sci. 1997;94(17):8954–8. 9256416 10.1073/pnas.94.17.8954 PMC22980

[108] Shi X, Zhang H, Lin S. Tandem repeats, high copy number and remarkable diel expression rhythm of form II RuBisCO in Prorocentrum donghaiense (Dinophyceae). PLoS ONE. 2013;8(8): e71232. 23976999 10.1371/journal.pone.0071232 PMC3747160

[109] Beauchemin M, Roy S, Daoust P, Dagenais-Bellefeuille S, Bertomeu T, Letourneau L, et al. Dinoflagellate tandem array gene transcripts are highly conserved and not polycistronic. Proc Natl Acad Sci. 2012;109(39):15793–8. 23019363 10.1073/pnas.1206683109 PMC3465430

[110] Stephens TG, Ragan MA, Bhattacharya D, Chan CX. Core genes in diverse dinoflagellate lineages include a wealth of conserved dark genes with unknown functions. Sci Rep. 2018;8(1):17175. 30464192 10.1038/s41598-018-35620-z PMC6249206

[111] Lin S, Litaker RW, Sunda WG. Phosphorus physiological ecology and molecular mechanisms in marine phytoplankton. J Phycol. 2016;52(1):10–36. 26987085 10.1111/jpy.12365

[112] Morey JS, Monroe EA, Kinney AL, Beal M, Johnson JG, Hitchcock GL, et al. Transcriptomic response of the red tide dinoflagellate, Karenia Brevis, to nitrogen and phosphorus depletion and addition. BMC Genomics. 2011;12:1–18. 10.1186/1471-2164-12-346 PMC3149589 21729317

[113] Lin X, Zhang H, Huang B, Lin S. Alkaline phosphatase gene sequence characteristics and transcriptional regulation by phosphate limitation in Karenia Brevis (Dinophyceae). Harmful Algae. 2012;17:14–24.

[114] Shi X, Ma M, Lin S. Cell cycle-dependent expression dynamics of G1/S specific cyclin, cellulose synthase and cellulase in the dinoflagellate Prorocentrum donghaiense. Front Microbiol. 2017;8: 1118. 28676796 10.3389/fmicb.2017.01118 PMC5476699

[115] Luo H, Lin X, Li L, Lin L, Zhang C, Lin S. Transcriptomic and physiological analyses of the dinoflagellate Karenia Mikimotoi reveal non-alkaline phosphatase‐based molecular machinery of ATP utilisation. Environ Microbiol. 2017;19(11):4506–18. 28856827 10.1111/1462-2920.13899

[116] Zhuang Y, Zhang H, Hannick L, Lin S. Metatranscriptome profiling reveals versatile N-nutrient utilization, CO2 limitation, oxidative stress, and active toxin production in an Alexandrium fundyense bloom. Harmful Algae. 2015;42:60–70.

[117] Okamoto OK, Hastings JW. Novel dinoflagellate clock-related genes identified through microarray analysis. J Phycol. 2003;39(3):519–26.

[118] Zaheri B, Morse D. An overview of transcription in dinoflagellates. Gene. 2022;829: 146505. 35447242 10.1016/j.gene.2022.146505

[119] Nikolov D, Burley S. RNA polymerase II transcription initiation: a structural view. Proc Natl Acad Sci. 1997;94(1):15–22. 8990153 10.1073/pnas.94.1.15 PMC33652

[120] Guillebault D, Sasorith S, Derelle E, Wurtz J-M, Lozano J-C, Bingham S, et al. A new class of transcription initiation factors, intermediate between TATA box-binding proteins (TBPs) and TBP-like factors (TLFs), is present in the marine unicellular organism, the dinoflagellate Crypthecodinium cohnii. J Biol Chem. 2002;277(43):40881–6. 12154093 10.1074/jbc.M205624200

[121] Sasaki K, Imai R. Pleiotropic roles of cold shock domain proteins in plants. Front Plant Sci. 2012;2:116. 22639630 10.3389/fpls.2011.00116 PMC3355641

[122] Beauchemin M, Roy S, Pelletier S, Averback A, Lanthier F, Morse D. Characterization of two dinoflagellate cold shock domain proteins. Msphere. 2016;1(1). 10.1128/msphere.00034–15 . PMC4863620 27303711

[123] Deng Y, Hu Z, Chai Z, Tang YZ. Cloning and partial characterization of a cold shock domain-containing protein gene from the Dinoflagellate Scrippsiella trochoidea. J Eukaryot Microbiol. 2019;66(3):393–403. 30099808 10.1111/jeu.12681

[124] Wang H, Kim H, Ki J-S. Transcriptomic identification and expression analysis of cold shock domain protein (CSP) genes in the marine dinoflagellate Prorocentrum minimum. J Appl Phycol. 2021;33(2):843–54.

[125] Baumgarten S, Bayer T, Aranda M, Liew YJ, Carr A, Micklem G, et al. Integrating microRNA and mRNA expression profiling in Symbiodinium microadriaticum, a dinoflagellate symbiont of reef-building corals. BMC Genomics. 2013;14:1–18. 24119094 10.1186/1471-2164-14-704 PMC3853145

[126] Moriano-Gutierrez S, Bongrand C, Essock-Burns T, Wu L, McFall-Ngai MJ, Ruby EG. The noncoding small RNA SsrA is released by Vibrio fischeri and modulates critical host responses. PLoS Biol. 2020;18(11): e3000934. 33141816 10.1371/journal.pbio.3000934 PMC7665748

[127] Moriano-Gutierrez S, Koch EJ, Bussan H, Romano K, Belcaid M, Rey FE, et al. Critical symbiont signals drive both local and systemic changes in diel and developmental host gene expression. Proc Natl Acad Sci. 2019;116(16):7990–9. 30833394 10.1073/pnas.1819897116 PMC6475425

[128] Weiberg A, Wang M, Lin F-M, Zhao H, Zhang Z, Kaloshian I, et al. Fungal small RNAs suppress plant immunity by hijacking host RNA interference pathways. Science. 2013;342(6154):118–23. 24092744 10.1126/science.1239705 PMC4096153

[129] Bowazolo C, Song B, Dorion S, Beauchemin M, Chevrier S, Rivoal J, et al. Orchestrated translation specializes dinoflagellate metabolism three times per day. Proc Natl Acad Sci. 2022;119(30): e2122335119. 35858433 10.1073/pnas.2122335119 PMC9335273

[130] Jones GD, Williams EP, Place AR, Jagus R, Bachvaroff TR. The alveolate translation initiation factor 4E family reveals a custom toolkit for translational control in core dinoflagellates. BMC Evol Biol. 2015;15:1–12. 25886308 10.1186/s12862-015-0301-9 PMC4330643

[131] Gray MW. Evolutionary origin of RNA editing. Biochemistry. 2012;51(26):5235–42. 22708551 10.1021/bi300419r

[132] Lin S, Zhang H, Spencer DF, Norman JE, Gray MW. Widespread and extensive editing of mitochondrial mRNAS in dinoflagellates. J Mol Biol. 2002;320(4):727–39. 12095251 10.1016/s0022-2836(02)00468-0

[133] Dang Y, Green BR. Substitutional editing of Heterocapsa triquetra chloroplast transcripts and a folding model for its divergent chloroplast 16S rRNA. Gene. 2009;442(1–2):73–80. 19376212 10.1016/j.gene.2009.04.006

[134] Mungpakdee S, Shinzato C, Takeuchi T, Kawashima T, Koyanagi R, Hisata K, et al. Massive gene transfer and extensive RNA editing of a symbiotic dinoflagellate plastid genome. Genome Biol Evol. 2014;6(6):1408–22. 24881086 10.1093/gbe/evu109 PMC4079212

[135] Shoguchi E, Yoshioka Y, Shinzato C, Arimoto A, Bhattacharya D, Satoh N. Correlation between organelle genetic variation and RNA editing in dinoflagellates associated with the coral Acropora digitifera. Genome Biol Evol. 2020;12(3):203–9. 32108224 10.1093/gbe/evaa042 PMC7144361

[136] Matsuo E, Morita K, Nakayama T, Yazaki E, Sarai C, Takahashi K, et al. Comparative plastid genomics of green-colored dinoflagellates unveils parallel genome compaction and RNA editing. Front Plant Sci. 2022;13: 918543. 35898209 10.3389/fpls.2022.918543 PMC9309888

[137] Dorrell RG, Howe CJ. Functional remodeling of RNA processing in replacement chloroplasts by pathways retained from their predecessors. Proc Natl Acad Sci. 2012;109(46):18879–84. 23112181 10.1073/pnas.1212270109 PMC3503182

[138] Jackson CJ, Gornik SG, Waller RF. A tertiary plastid gains RNA editing in its new host. Mol Biol Evol. 2013;30(4):788–92. 23197592 10.1093/molbev/mss270

[139] He J, Huang Y, Li L, Lin S, Ma M, Wang Y, et al. Novel plastid genome characteristics in Fugacium Kawagutii and the trend of accelerated evolution of plastid proteins in dinoflagellates. Genome Biol Evol. 2024;16(1): evad237. 38155596 10.1093/gbe/evad237 PMC10781511

[140] Wiese M, Murray SA, Alvin A, Neilan BA. Gene expression and molecular evolution of sxtA4 in a saxitoxin producing dinoflagellate Alexandrium catenella. Toxicon. 2014;92:102–12. 25301480 10.1016/j.toxicon.2014.09.015

[141] Curtis BA, Tanifuji G, Burki F, Gruber A, Irimia M, Maruyama S, et al. Algal genomes reveal evolutionary mosaicism and the fate of nucleomorphs. Nature. 2012;492(7427):59–65. 23201678 10.1038/nature11681

[142] Sarai C, Tanifuji G, Nakayama T, Kamikawa R, Takahashi K, Yazaki E, et al. Dinoflagellates with relic endosymbiont nuclei as models for elucidating organellogenesis. Proc Natl Acad Sci. 2020;117(10):5364–75. 32094181 10.1073/pnas.1911884117 PMC7071878

[143] Zhang Z, Green B, Cavalier-Smith T. Single gene circles in dinoflagellate chloroplast genomes. Nature. 1999;400(6740):155–9. 10408440 10.1038/22099

[144] Imanian B, Pombert J-F, Keeling PJ. The complete plastid genomes of the two ‘dinotoms’ Durinskia Baltica and Kryptoperidinium foliaceum. PLoS ONE. 2010;5(5):e10711. 20502706 10.1371/journal.pone.0010711 PMC2873285

[145] Hofmann E, Wrench PM, Sharples FP, Hiller RG, Welte W, Diederichs K. Structural basis of light harvesting by carotenoids: peridinin-chlorophyll-protein from Amphidinium carterae. Science. 1996;272(5269):1788–91. 8650577 10.1126/science.272.5269.1788

[146] Jiang J, Zhang H, Orf GS, Lu Y, Xu W, Harrington LB, et al. Evidence of functional trimeric chlorophyll a/c2-peridinin proteins in the dinoflagellate Symbiodinium. Biochim et Biophys Acta (BBA)-Bioenergetics. 2014;1837(11):1904–12. 10.1016/j.bbabio.2014.07.023 25150185

[147] Sharples FP, Wrench PM, Ou K, Hiller RG. Two distinct forms of the peridinin-chlorophyll a-protein from Amphidinium carterae. Biochim et Biophys Acta (BBA)-Bioenergetics. 1996;1276(2):117–23. 10.1016/0005-2728(96)00066-7 8816945

[148] Ilagan RP, Koscielecki JF, Hiller RG, Sharples FP, Gibson GN, Birge RR, et al. Femtosecond Time-resolved absorption spectroscopy of main-form and high-salt peridinin – chlorophyll a – proteins at low temperatures. Biochemistry. 2006;45(47):14052–63. 17115700 10.1021/bi061217u

[149] Lin S, Wu S, He J, Wang X, Grossman AR. Shining light on dinoflagellate photosystem I. Nat Commun. 2024;15(1):3337. 38637576 10.1038/s41467-024-47797-1 PMC11026431

[150] Fromme P, Jordan P, Krauß N. Structure of photosystem I. Biochim et Biophys Acta (BBA)-Bioenergetics. 2001;1507(1–3):5–31. 10.1016/s0005-2728(01)00195-5 11687205

[151] Pi X, Tian L, Dai H-E, Qin X, Cheng L, Kuang T, et al. Unique organization of photosystem I–light-harvesting supercomplex revealed by cryo-EM from a red alga. Proc Natl Acad Sci. 2018;115(17):4423–8. 29632169 10.1073/pnas.1722482115 PMC5924924

[152] Qin X, Pi X, Wang W, Han G, Zhu L, Liu M, et al. Structure of a green algal photosystem I in complex with a large number of light-harvesting complex I subunits. Nat Plants. 2019;5(3):263–72. 30850820 10.1038/s41477-019-0379-y

[153] Gisriel CJ, Brudvig GW. Comparison of PsbQ and Psb27 in photosystem II provides insight into their roles. Photosynth Res. 2022;152(2):177–91. 35001227 10.1007/s11120-021-00888-2 PMC9271139

[154] Su X, Ma J, Pan X, Zhao X, Chang W, Liu Z, et al. Antenna arrangement and energy transfer pathways of a green algal photosystem-I–LHCI supercomplex. Nat Plants. 2019;5(3):273–81. 30850819 10.1038/s41477-019-0380-5

[155] Su X, Cao D, Pan X, Shi L, Liu Z, Dall’Osto L, et al. Supramolecular assembly of chloroplast NADH dehydrogenase-like complex with photosystem I from Arabidopsis thaliana. Mol Plant. 2022;15(3):454–67. 35123031 10.1016/j.molp.2022.01.020

[156] Zhao S, Shen L, Li X, Tao Q, Li Z, Xu C, et al. Structural insights into photosystem II supercomplex and trimeric FCP antennae of a centric diatom Cyclotella meneghiniana. Nat Commun. 2023;14(1):8164. 38071196 10.1038/s41467-023-44055-8 PMC10710467

[157] Kato H, Tokutsu R, Kubota-Kawai H, Burton-Smith RN, Kim E, Minagawa J. Characterization of a giant PSI supercomplex in the symbiotic dinoflagellate Symbiodiniaceae. Plant Physiol. 2020;183(4):1725–34. 32546570 10.1104/pp.20.00726 PMC7401106

[158] Zhao L-S, Wang N, Li K, Li C-Y, Guo J-P, He F-Y, et al. Architecture of symbiotic dinoflagellate photosystem I–light-harvesting supercomplex in Symbiodinium. Nat Commun. 2024;15(1):2392. 38493166 10.1038/s41467-024-46791-x PMC10944487

[159] Slavov C, Schrameyer V, Reus M, Ralph PJ, Hill R, Büchel C, et al. Super-quenching state protects Symbiodinium from thermal stress—implications for coral bleaching. Biochim Et Biophys Acta (BBA)-Bioenergetics. 2016;1857(6):840–7. 26869375 10.1016/j.bbabio.2016.02.002

[160] Gong S, Jin X, Xiao Y, Li Z. Ocean acidification and warming lead to increased growth and altered chloroplast morphology in the thermo-tolerant alga Symbiochlorum Hainanensis. Front Plant Sci. 2020;11: 585202. 33281847 10.3389/fpls.2020.585202 PMC7705064

[161] Sandkvist M. Type II secretion and pathogenesis. Infect Immun. 2001;69(6):3523–35. 11349009 10.1128/IAI.69.6.3523-3535.2001 PMC98326

[162] Cianciotto NP, White RC. Expanding role of type II secretion in bacterial pathogenesis and beyond. Infect Immun. 2017;85(5):00014–7. 10.1128/iai. 10.1128/IAI.00014-17 PMC5400843 28264910

[163] Epstein B, Tiffin P. Comparative genomics reveals high rates of horizontal transfer and strong purifying selection on rhizobial symbiosis genes. Proc Royal Soc B. 2021;288(1942):20201804. 10.1098/rspb.2020.1804 PMC7892418 33402066

[164] Banaszak AT, LaJeunesse TC, Trench RK. The synthesis of mycosporine-like amino acids (MAAs) by cultured, symbiotic dinoflagellates. J Exp Mar Biol Ecol. 2000;249(2):219–33. 10841936 10.1016/s0022-0981(00)00192-1

[165] Wang L, Lin X, Goes JI, Lin S. Phylogenetic analyses of three genes of Pedinomonas noctilucae, the green endosymbiont of the marine dinoflagellate Noctiluca scintillans, reveal its affiliation to the order Marsupiomonadales (Chlorophyta, Pedinophyceae) under the reinstated name Protoeuglena noctilucae. Protist. 2016;167(2):205–16. 27033730 10.1016/j.protis.2016.02.005

[166] Luo H, Wang J, Goes JI, Gomes HdR, Al-Hashmi K, Tobias C, et al. A grazing-driven positive nutrient feedback loop and active sexual reproduction underpin widespread Noctiluca green tides. ISME Commun. 2022;2(1):103. 37938758 10.1038/s43705-022-00187-4 PMC9723592

[167] Mao X, Chen J, van Oosterhout C, Zhang H, Liu G, Zhuang Y, et al. Diversity, prevalence, and expression of cyanase genes (cynS) in planktonic marine microorganisms. ISME J. 2022;16(2):602–5. 34408267 10.1038/s41396-021-01081-y PMC8776842

[168] Kopp C, Pernice M, Domart-Coulon I, Djediat C, Spangenberg JE, Alexander DT, et al. Highly dynamic cellular-level response of symbiotic coral to a sudden increase in environmental nitrogen. MBio. 2013;4(3):00052–13. 10.1128/mbio. 10.1128/mBio.00052-13 PMC3656441 23674611

[169] Jantschke A, Pinkas I, Hirsch A, Elad N, Schertel A, Addadi L, et al. Anhydrous β-guanine crystals in a marine dinoflagellate: structure and suggested function. J Struct Biol. 2019;207(1):12–20. 30991101 10.1016/j.jsb.2019.04.009

[170] Mojzeš P, Gao L, Ismagulova T, Pilátová J, Moudříková Š, Gorelova O, et al. Guanine, a high-capacity and rapid-turnover nitrogen reserve in microalgal cells. Proc Natl Acad Sci. 2020;117(51):32722–30. 33293415 10.1073/pnas.2005460117 PMC7768779

[171] McKie-Krisberg ZM, Sanders RW, Gast RJ. Evaluation of Mixotrophy-Associated Gene expression in two species of Polar Marine Algae. Front Mar Sc. 2018;5:5.

[172] Uribe-Querol E, Rosales C. Phagocytosis: Our current understanding of a Universal Biological process. Front Immunol. 2020;11:1066. 32582172 10.3389/fimmu.2020.01066 PMC7280488

[173] Li H, Chen J, Yu L, Fan G, Li T, Li L, et al. In situ community transcriptomics illuminates CO2-fixation potentials and supporting roles of phagotrophy and proton pump in plankton in a subtropical marginal sea. Microbiol Spectr. 2024;12(3):e02177-02123. 38319114 10.1128/spectrum.02177-23 PMC10913738

[174] Mitra A, Flynn KJ, Tillmann U, Raven JA, Caron D, Stoecker DK, et al. Defining planktonic protist functional groups on mechanisms for energy and nutrient acquisition: incorporation of diverse mixotrophic strategies. Protist. 2016;167(2):106–20. 26927496 10.1016/j.protis.2016.01.003

[175] Villanova V, Spetea C. Mixotrophy in diatoms: molecular mechanism and industrial potential. Physiol Plant. 2021;173(2):603–11. 34076276 10.1111/ppl.13471

[176] Stoecker DK. Mixotrophy among dinoflagellates 1. J Eukaryot Microbiol. 1999;46(4):397–401.

[177] Jeong HJ, Yoo YD, Kim JS, Seong KA, Kang NS, Kim TH. Growth, feeding and ecological roles of the mixotrophic and heterotrophic dinoflagellates in marine planktonic food webs. Ocean Sci J. 2010;45:65–91.

[178] Yu L, Li T, Li H, Ma M, Li L, Lin S. In situ molecular ecological analyses illuminate distinct factors regulating formation and demise of a harmful dinoflagellate bloom. Microbiol Spectr. 2023;11(3):e05157-05122. 37074171 10.1128/spectrum.05157-22 PMC10269597

[179] Kellmann R, Stüken A, Orr RJ, Svendsen HM, Jakobsen KS. Biosynthesis and molecular genetics of polyketides in marine dinoflagellates. Mar Drugs. 2010;8(4):1011–48. 20479965 10.3390/md8041011 PMC2866473

[180] Hertweck C. The biosynthetic logic of polyketide diversity. Angew Chem Int Ed. 2009;48(26):4688–716. 10.1002/anie.200806121 19514004

[181] Marahiel MA, Stachelhaus T, Mootz HD. Modular peptide synthetases involved in nonribosomal peptide synthesis. Chem Rev. 1997;97(7):2651–74. 11851476 10.1021/cr960029e

[182] López-Legentil S, Song B, DeTure M, Baden DG. Characterization and localization of a hybrid non-ribosomal peptide synthetase and polyketide synthase gene from the toxic dinoflagellate Karenia Brevis. Mar Biotechnol. 2010;12:32–41. 10.1007/s10126-009-9197-y 19468793

[183] Bachvaroff TR, Williams E, Jagus R, Place AR. A noncryptic noncanonical multi-module PKS/NRPS found in dinoflagellates. In: Proceedings of the 16th International Conference on Harmful Algae. Wellington: Cawthron Institute, Nelson, New Zealand and; 2015. PMC6755914 31549100

[184] Verma A, Kohli GS, Harwood DT, Ralph PJ, Murray SA. Transcriptomic investigation into polyketide toxin synthesis in Ostreopsis (Dinophyceae) species. Environ Microbiol. 2019;21(11):4196–211. 31415128 10.1111/1462-2920.14780

[185] Van Dolah FM, Morey JS, Milne S, Ung A, Anderson PE, Chinain M. Transcriptomic analysis of polyketide synthases in a highly ciguatoxic dinoflagellate, Gambierdiscus polynesiensis and low toxicity Gambierdiscus Pacificus, from French polynesia. PLoS ONE. 2020;15(4):e0231400. 32294110 10.1371/journal.pone.0231400 PMC7159223

[186] Beedessee G, Hisata K, Roy MC, Van Dolah FM, Satoh N, Shoguchi E. Diversified secondary metabolite biosynthesis gene repertoire revealed in symbiotic dinoflagellates. Sci Rep. 2019;9(1):1204. 30718591 10.1038/s41598-018-37792-0 PMC6361889

[187] Monroe EA, Van Dolah FM. The toxic dinoflagellate Karenia Brevis encodes novel type I-like Polyketide synthases Containing Discrete Catalytic domains. Protist. 2008;159(3):471–82. 18467171 10.1016/j.protis.2008.02.004

[188] Van Dolah FM, Kohli GS, Morey JS, Murray SA. Both modular and single-domain type I polyketide synthases are expressed in the brevetoxin‐producing dinoflagellate, Karenia Brevis (Dinophyceae). J Phycol. 2017;53(6):1325–39. 28949419 10.1111/jpy.12586 PMC5725682

[189] Kohli GS, John U, Van Dolah FM, Murray SA. Evolutionary distinctiveness of fatty acid and polyketide synthesis in eukaryotes. ISME J. 2016;10(8):1877–90. 26784357 10.1038/ismej.2015.263 PMC5029157

[190] Williams EP, Bachvaroff TR, Place AR. A Global Approach to estimating the abundance and duplication of Polyketide Synthase domains in Dinoflagellates. Evolutionary Bioinf. 2021;17:11769343211031871. 10.1177/11769343211031871 PMC8283056 34345159

[191] Singh K, Winter M, Zouhar M, Ryšánek P. Cyclophilins: less studied proteins with critical roles in pathogenesis. Phytopathology. 2018;108(1):6–14. 28643580 10.1094/PHYTO-05-17-0167-RVW

[192] Singh H, Kaur K, Singh M, Kaur G, Singh P. Plant cyclophilins: multifaceted proteins with versatile roles. Front Plant Sci. 2020;11:585212. 33193535 10.3389/fpls.2020.585212 PMC7641896

[193] Vallon O. Chlamydomonas immunophilins and parvulins: survey and critical assessment of gene models. Eukaryot Cell. 2005;4(2):230–41. 15701785 10.1128/EC.4.2.230-241.2005 PMC549346

[194] Wu T-M, Hsu Y-T, Sung M-S, Hsu Y-T, Lee T-M. Expression of genes involved in redox homeostasis and antioxidant defense in a marine macroalga Ulva fasciata by excess copper. Aquat Toxicol. 2009;94(4):275–85. 19665240 10.1016/j.aquatox.2009.07.010

[195] Ponmani T, Guo R, Ki J-S. A novel cyclophilin gene from the dinoflagellate Prorocentrum minimum and its possible role in the environmental stress response. Chemosphere. 2015;139:260–7. 26150195 10.1016/j.chemosphere.2015.06.036

[196] Abassi S, Wang H, Park BS, Park J-W, Ki J-SA, Novel Cyclophilin. B gene in the Red Tide Dinoflagellate Cochlodinium polykrikoides: molecular characterizations and transcriptional responses to environmental stresses. Biomed Res Int. 2017;2017(1):4101580. 29226135 10.1155/2017/4101580 PMC5684524

[197] Calandra T, Roger T. Macrophage migration inhibitory factor: a regulator of innate immunity. Nat Rev Immunol. 2003;3(10):791–800. 14502271 10.1038/nri1200 PMC7097468

[198] Michelet C, Danchin EG, Jaouannet M, Bernhagen J, Panstruga R, Kogel K-H, et al. Cross-kingdom analysis of diversity, evolutionary history, and site selection within the eukaryotic macrophage migration inhibitory factor superfamily. Genes. 2019;10(10):740. 31554205 10.3390/genes10100740 PMC6826473

[199] Jung H, Seong H-A, Ha H. Critical role of cysteine residue 81 of macrophage migration inhibitory factor (MIF) in MIF-induced inhibition of p53 activity. J Biol Chem. 2008;283(29):20383–96. 18502749 10.1074/jbc.M800050200

[200] Augustijn KD, Kleemann R, Thompson J, Kooistra T, Crawford CE, Reece SE, et al. Functional characterization of the Plasmodium Falciparum and P. Berghei homologues of macrophage migration inhibitory factor. Infect Immun. 2007;75(3):1116–28. 17158894 10.1128/IAI.00902-06 PMC1828592

[201] Miller JL, Harupa A, Kappe SH, Mikolajczak SA. Plasmodium Yoelii macrophage migration inhibitory factor is necessary for efficient liver-stage development. Infect Immun. 2012;80(4):1399–407. 22252874 10.1128/IAI.05861-11 PMC3318411

[202] Ajonina-Ekoti I, Kurosinski MA, Younis AE, Ndjonka D, Tanyi MK, Achukwi M, et al. Comparative analysis of macrophage migration inhibitory factors (MIFs) from the parasitic nematode Onchocerca Volvulus and the free-living nematode Caenorhabditis elegans. Parasitol Res. 2013;112:3335–46. 23820606 10.1007/s00436-013-3513-1

[203] Naessens E, Dubreuil G, Giordanengo P, Baron OL, Minet-Kebdani N, Keller H, et al. A secreted MIF cytokine enables aphid feeding and represses plant immune responses. Curr Biol. 2015;25(14):1898–903. 26119751 10.1016/j.cub.2015.05.047

[204] Wasiel AA, Rozeboom HJ, Hauke D, Baas B-J, Zandvoort E, Quax WJ, et al. Structural and functional characterization of a Macrophage Migration Inhibitory factor Homologue from the Marine Cyanobacterium Prochlorococcus marinus. Biochemistry. 2010;49(35):7572–81. 20715791 10.1021/bi1008276

[205] Jaouannet M, Pavaux A-S, Pagnotta S, Pierre O, Michelet C, Marro S, et al. Atypical membrane-anchored Cytokine MIF in a Marine Dinoflagellate. Microorganisms. 2020;8(9): 1263. 32825358 10.3390/microorganisms8091263 PMC7565538

[206] Islas-Flores T, Galán-Vásquez E, Villanueva MA. Screening a spliced leader-based Symbiodinium microadriaticum cDNA library using the yeast-two hybrid system reveals a hemerythrin-like protein as a putative SmicRACK1 ligand. Microorganisms. 2021;9(4):791. 33918967 10.3390/microorganisms9040791 PMC8070245

[207] Adams DR, Ron D, Kiely PA. RACK1, a multifaceted scaffolding protein: structure and function. Cell Communica Signal. 2011;9:1–24. 10.1186/1478-811X-9-22 PMC3195729 21978545

[208] Li X, Li J, Hu X, Huang L, Xiao J, Chan J, et al. Differential roles of the hemerythrin-like proteins of Mycobacterium smegmatis in hydrogen peroxide and erythromycin susceptibility. Sci Rep. 2015;5(1): 16130. 26607739 10.1038/srep16130 PMC4660385

[209] Alvarez-Carreno C, Becerra A, Lazcano A. Molecular evolution of the oxygen-binding hemerythrin domain. PLoS ONE. 2016;11(6): e0157904. 27336621 10.1371/journal.pone.0157904 PMC4919030

[210] Coates CJ, Decker H. Immunological properties of oxygen-transport proteins: hemoglobin, hemocyanin and hemerythrin. Cell Mol Life Sci. 2017;74:293–317. 27518203 10.1007/s00018-016-2326-7 PMC5219038

[211] Krochko JE, Abrams GD, Loewen MK, Abrams SR, Cutler AJ. (+)-Abscisic acid 8′-hydroxylase is a cytochrome P450 monooxygenase. Plant Physiol. 1998;118(3):849–60. 9808729 10.1104/pp.118.3.849 PMC34795

[212] Footitt S, Douterelo-Soler I, Clay H, Finch-Savage WE. Dormancy cycling in Arabidopsis seeds is controlled by seasonally distinct hormone-signaling pathways. Proc Natl Acad Sci. 2011;108(50):20236–41. 22128331 10.1073/pnas.1116325108 PMC3250134

[213] Deng Y, Hu Z, Shang L, Peng Q, Tang YZ. Transcriptomic analyses of Scrippsiella trochoidea reveals processes regulating encystment and dormancy in the life cycle of a Dinoflagellate, with a Particular attention to the role of Abscisic Acid. Front Microbiol. 2017;8:8. 29312167 10.3389/fmicb.2017.02450 PMC5732363

[214] Rosic NN, Pernice M, Dunn S, Dove S, Hoegh-Guldberg O. Differential regulation by heat stress of novel cytochrome P450 genes from the dinoflagellate symbionts of reef-building corals. Appl Environ Microbiol. 2010;76(9):2823–9. 20228102 10.1128/AEM.02984-09 PMC2863455

[215] Haslbeck M, Vierling E. A first line of stress defense: small heat shock proteins and their function in protein homeostasis. J Mol Biol. 2015;427(7):1537–48. 25681016 10.1016/j.jmb.2015.02.002 PMC4360138

[216] Haslbeck M, Weinkauf S, Buchner J. Small heat shock proteins: simplicity meets complexity. J Biol Chem. 2019;294(6):2121–32. 30385502 10.1074/jbc.REV118.002809 PMC6369295

[217] Waters ER, Vierling E. Plant small heat shock proteins–evolutionary and functional diversity. New Phytol. 2020;227(1):24–37. 32297991 10.1111/nph.16536

[218] Deng Y, Li F, Hu Z, Yue C, Tang YZ. The implication inferred from the expression of small heat-shock protein genes in dinoflagellate resting cysts buried in Marine Sediment. Diversity. 2021;13(10):471.

[219] Vesteg M, Krajčovič J, editors. On the origin of meiosis and sex. Biology Forum/Rivista di Biologia 2007;100(1):147-61. 17592823

[220] Goodenough U, Heitman J. Origins of eukaryotic sexual reproduction. Cold Spring Harb Perspect Biol. 2014;6(3): a016154. 24591519 10.1101/cshperspect.a016154 PMC3949356

[221] Speijer D, Lukeš J, Eliáš M. Sex is a ubiquitous, ancient, and inherent attribute of eukaryotic life. Proc Natl Acad Sci. 2015;112(29):8827–34. 26195746 10.1073/pnas.1501725112 PMC4517231

[222] Morse D. A transcriptome-based perspective of meiosis in dinoflagellates. Protist. 2019;170(4):397–403. 31521988 10.1016/j.protis.2019.06.003

[223] Chi J, Parrow MW, Dunthorn M. Cryptic sex in Symbiodinium (Alveolata, Dinoflagellata) is supported by an inventory of meiotic genes. J Eukaryot Microbiol. 2014;61(3):322–7. 24904932 10.1111/jeu.12110

[224] Schurko AM, Logsdon JM Jr. Using a meiosis detection toolkit to investigate ancient asexual “scandals” and the evolution of sex. BioEssays. 2008;30(6):579–89. 18478537 10.1002/bies.20764

[225] Shah S, Chen Y, Bhattacharya D, Chan CX. Sex in Symbiodiniaceae dinoflagellates: genomic evidence for independent loss of the canonical synaptonemal complex. Sci Rep. 2020;10(1):9792. 32555361 10.1038/s41598-020-66429-4 PMC7299967

[226] Berdieva MA, Pozdnyakov IA, Kalinina VO, Skarlato SO. Putative meiotic toolkit in the dinoflagellate Prorocentrum cordatum: additional evidence for sexual process from transcriptome. J Eukaryot Microbiol. 2021;68(3):e12845. 33624379 10.1111/jeu.12845

[227] Wong JL, Johnson MA. Is HAP2-GCS1 an ancestral gamete fusogen? Trends Cell Biol. 2010;20(3):134–41. 20080406 10.1016/j.tcb.2009.12.007

[228] Bravo I, Figueroa RI. Towards an ecological understanding of Dinoflagellate Cyst functions. Microorganisms. 2014;2(1):11–32. 27694774 10.3390/microorganisms2010011 PMC5029505

[229] Figueroa RI, Bravo I, Garcés E. The significance of sexual versus asexual cyst formation in the life cycle of the noxious dinoflagellate Alexandrium Peruvianum. Harmful Algae. 2008;7(5):653–63.

[230] Zhang H, Gu B, Zhou Y, Ma X, Liu T, Xu H, et al. Multi-omics profiling reveals resource allocation and acclimation strategies to temperature changes in a marine dinoflagellate. Appl Environ Microbiol. 2022;88(17):e01213-01222. 35976001 10.1128/aem.01213-22 PMC9469709

[231] Margulies E, White A, Sherry S. Sulfinpyrazone: a review of its pharmacological properties and therapeutic use. Drugs. 1980;20:179–97. 7000488 10.2165/00003495-198020030-00002

[232] Gornik SG, Maegele I, Hambleton EA, Voss PA, Waller RF, Guse A. Nuclear transformation of a dinoflagellate symbiont of corals. Front Mar Sci. 2022;9:1035413.

[233] Nimmo IC, Barbrook AC, Lassadi I, Chen JE, Geisler K, Smith AG, et al. Genetic transformation of the dinoflagellate chloroplast. Elife. 2019;8: e45292. 31317866 10.7554/eLife.45292 PMC6639071

[234] Sprecher BN, Zhang H, Lin S. Nuclear gene transformation in a dinoflagellate. bioRxiv. 2019:602821. 10.3390/microorganisms8010126 PMC7022241 31963386

[235] Chan WS, Kwok ACM, Wong JTY. Knockdown of dinoflagellate cellulose synthase CesA1 resulted in malformed intracellular cellulosic thecal plates and severely impeded cyst-to-swarmer transition. Front Microbiol. 2019;10:546. 30941114 10.3389/fmicb.2019.00546 PMC6433935

[236] Judd M, Place AR. A strategy for Gene Knockdown in dinoflagellates. Microorganisms. 2022;10(6): 1131. 35744649 10.3390/microorganisms10061131 PMC9228228

[237] Ciobanu D, Clum A, Ahrendt S, Andreopoulos WB, Salamov A, Chan S, et al. A single-cell genomics pipeline for environmental microbial eukaryotes. Iscience. 2021;24(4):102290. 33870123 10.1016/j.isci.2021.102290 PMC8042348

[238] Cooney EC, Okamoto N, Cho A, Hehenberger E, Richards TA, Santoro AE, et al. Single-cell transcriptomics of Abedinium reveals a new early-branching dinoflagellate lineage. Genome Biol Evol. 2020;12(12):2417–28. 33045041 10.1093/gbe/evaa196 PMC7846120

[239] Ma M, Li H, Wang C, Li T, Wang J, Yuan H, et al. A comparative study reveals the relative importance of prokaryotic and eukaryotic proton pump rhodopsins in a subtropical marginal sea. ISME Commun. 2023;3(1):79. 37596487 10.1038/s43705-023-00292-y PMC10439184

